# Structure of a Bimodular Botulinum Neurotoxin Complex Provides Insights into Its Oral Toxicity

**DOI:** 10.1371/journal.ppat.1003690

**Published:** 2013-10-10

**Authors:** Kwangkook Lee, Shenyan Gu, Lei Jin, Thi Tuc Nghi Le, Luisa W. Cheng, Jasmin Strotmeier, Anna Magdalena Kruel, Guorui Yao, Kay Perry, Andreas Rummel, Rongsheng Jin

**Affiliations:** 1 Department of Physiology and Biophysics, University of California, Irvine, California, United States of America; 2 Infectious and Inflammatory Disease Center, Sanford-Burnham Medical Research Institute, La Jolla, California, United States of America; 3 Institut für Toxikologie, Medizinische Hochschule Hannover, Hannover, Germany; 4 Foodborne Contaminants Research Unit, Western Regional Research Center, United States Department of Agriculture, Agricultural Research Service, Albany, California, United States of America; 5 NE-CAT and Department of Chemistry and Chemical Biology, Cornell University, Argonne National Laboratory, Argonne, Illinois, United States of America; 6 Neuroscience, Aging and Stem Cell Center, Sanford-Burnham Medical Research Institute, La Jolla, California, United States of America; The Rockefeller University, United States of America

## Abstract

Botulinum neurotoxins (BoNTs) are produced by *Clostridium botulinum* and cause the fatal disease botulism, a flaccid paralysis of the muscle. BoNTs are released together with several auxiliary proteins as progenitor toxin complexes (PTCs) to become highly potent oral poisons. Here, we report the structure of a ∼760 kDa 14-subunit large PTC of serotype A (L-PTC/A) and reveal insight into its absorption mechanism. Using a combination of X-ray crystallography, electron microscopy, and functional studies, we found that L-PTC/A consists of two structurally and functionally independent sub-complexes. A hetero-dimeric 290 kDa complex protects BoNT, while a hetero-dodecameric 470 kDa complex facilitates its absorption in the harsh environment of the gastrointestinal tract. BoNT absorption is mediated by nine glycan-binding sites on the dodecameric sub-complex that forms multivalent interactions with carbohydrate receptors on intestinal epithelial cells. We identified monosaccharides that blocked oral BoNT intoxication in mice, which suggests a new strategy for the development of preventive countermeasures for BoNTs based on carbohydrate receptor mimicry.

## Author Summary

Food-borne botulinum neurotoxin (BoNT) poisoning results in fatal muscle paralysis. But how can BoNT–a large protein released by the bacteria clostridia–survive the hostile gastrointestinal (GI) tract to gain access to neurons that control muscle contraction? Here, we report the complete structure of a bimodular ∼760 kDa BoNT/A large progenitor toxin complex (L-PTC), which is composed of BoNT and four non-toxic bacterial proteins. The architecture of this bacterial machinery mimics an Apollo lunar module, whereby the “ascent stage” (a ∼290 kDa module) protects BoNT from destruction in the GI tract and the 3-arm “descent stage” (a ∼470 kDa module) mediates absorption of BoNT by binding to host carbohydrate receptors in the small intestine. This new finding has helped us identify the carbohydrate-binding sites and the monosaccharide IPTG as a prototypical oral inhibitor, which extends survival following lethal BoNT/A intoxication of mice. Hence, pre-treatment with small molecule inhibitors based on carbohydrate receptor mimicry can provide temporary protection against BoNT entry into the circulation.

## Introduction

The seven botulinum neurotoxin serotypes (BoNT/A–G) produced by *Clostridium botulinum* are the causative agents of the neuroparalytic syndrome of botulism and pose a serious threat for bioterrorism [1]. Conversely, BoNT/A is a highly effective therapy for treating neurological disorders [2]. The naturally occurring BoNTs are released together with up to four non-toxic neurotoxin-associated proteins (NAPs) (also called associated non-toxic proteins, ANTPs) in the form of progenitor toxin complexes (PTCs) with different molecular compositions [3]. Such PTCs are highly potent food poisons, e.g., the PTC of BoNT/A displays an oral LD_50_ of ∼35 µg/kg body weight [4]. While BoNT is sensitive to denaturation by the acidic environment and digestive proteases present in the gastrointestinal (GI) tract [5], the PTCs of different serotypes exhibit ∼360–16,000-fold greater oral toxicity than free BoNT [4], [6], [7], [8]. The NAPs are encoded together with the *bont* gene in one of two different gene clusters, the HA cluster or the orfX cluster [9]. Both clusters encode the non-toxic non-hemagglutinin (NTNHA) protein, which adopts a BoNT-like structure despite its lack of neurotoxicity [5]. The HA gene cluster also encodes three hemagglutinins (HA70, HA17, and HA33; also called HA3, HA2, and HA1, respectively), which together with BoNT and NTNHA constitute the large PTC (L-PTC) [10]. The structure and function of the corresponding orfX proteins are largely unknown [11].

Structural information of HAs is available for serotypes C and D, such as the crystal structures of HA33 of serotype C (HA33-C) [12], [13], a complex composed of HA17 and HA33 of serotype D [14], and HA70 of serotype C (HA70-C) [15], [16]. However, BoNT/C and D rarely cause human botulism but are known to cause the syndrome in cattle, poultry, and wild birds. For BoNT/A, the major cause of human botulism, only the structure of HA33 (HA33-A), which displays an amino-acid identity of ∼38% to HA33-C and D, has been solved [17]. We have recently determined the crystal structure of the BoNT/A–NTNHA complex [5]. However, it remains largely unclear how the HAs assemble with one another and how they interact with BoNT and NTNHA.

Various structural models have been proposed for the L-PTC. One recent paper suggested a complex composed of BoNT∶NTNHA∶HA70∶HA17∶HA33 in a 1∶1∶2∶2∶3 ratio for L-PTC/A [18], whereas earlier studies suggested a stoichiometry of 1∶1∶3–5∶5–6∶8–9 or 1∶1∶3∶3∶4 for L-PTC/A, or 1∶1∶2∶4∶4 for L-PTC/D [19], [20], [21]. In comparison, electron microscopy (EM) studies on L-PTC/A, B and D supported a stoichiometry of 1∶1∶3∶3∶6 [14], [22].

The functional roles of NAPs are also not well defined. We have recently shown that NTNHA shields BoNT against low-pH denaturation and proteolytic attack in the GI tract by forming the minimally functional PTC (M-PTC), and releases it during entry into the general circulation [5], [23]. However, it is not clear whether HAs further protect the toxin. At the same time, the L-PTC may contribute to BoNT internalization into the host bloodstream through interactions with intestinal cell surface glycans [24], [25], [26]. The HAs of BoNT/A and B could disrupt the human epithelial intercellular junction through species-specific interaction with E-cadherin, presumably facilitating BoNT transport via the paracellular route [27], [28], [29]. Defining the L-PTC structure would permit a more complete understanding of the complex's role in toxin shielding and delivery, and would help to describe the molecular mechanism underlying these important actions.

Here, we report the structure of a ∼760 kDa L-PTC/A using a combination of X-ray crystallography, single-particle EM and three-dimensional reconstruction (3D-EM). We found that L-PTC/A consists of two structurally and functionally independent sub-complexes, the M-PTC and the HA complex. The HA complex is composed of HA70, HA17, and HA33 in a 3∶3∶6 stoichiometry and adopts an extended three-blade architecture, whereas the M-PTC is situated on top of the HA complex platform. BoNT/A absorption is mainly mediated by nine glycan-binding sites on the HA complex that together form multivalent interactions with host carbohydrate receptors on intestinal epithelial cells. HA complex-mediated toxin absorption can be blocked *in vitro* by carbohydrate receptor mimics. The monosaccharide IPTG also inhibits oral BoNT/A intoxication in mice, providing the first approach for a possible preventive treatment prior to deliberate BoNT poisoning.

## Results

### Electron microscopy structure of the L-PTC

**Figure 1:**
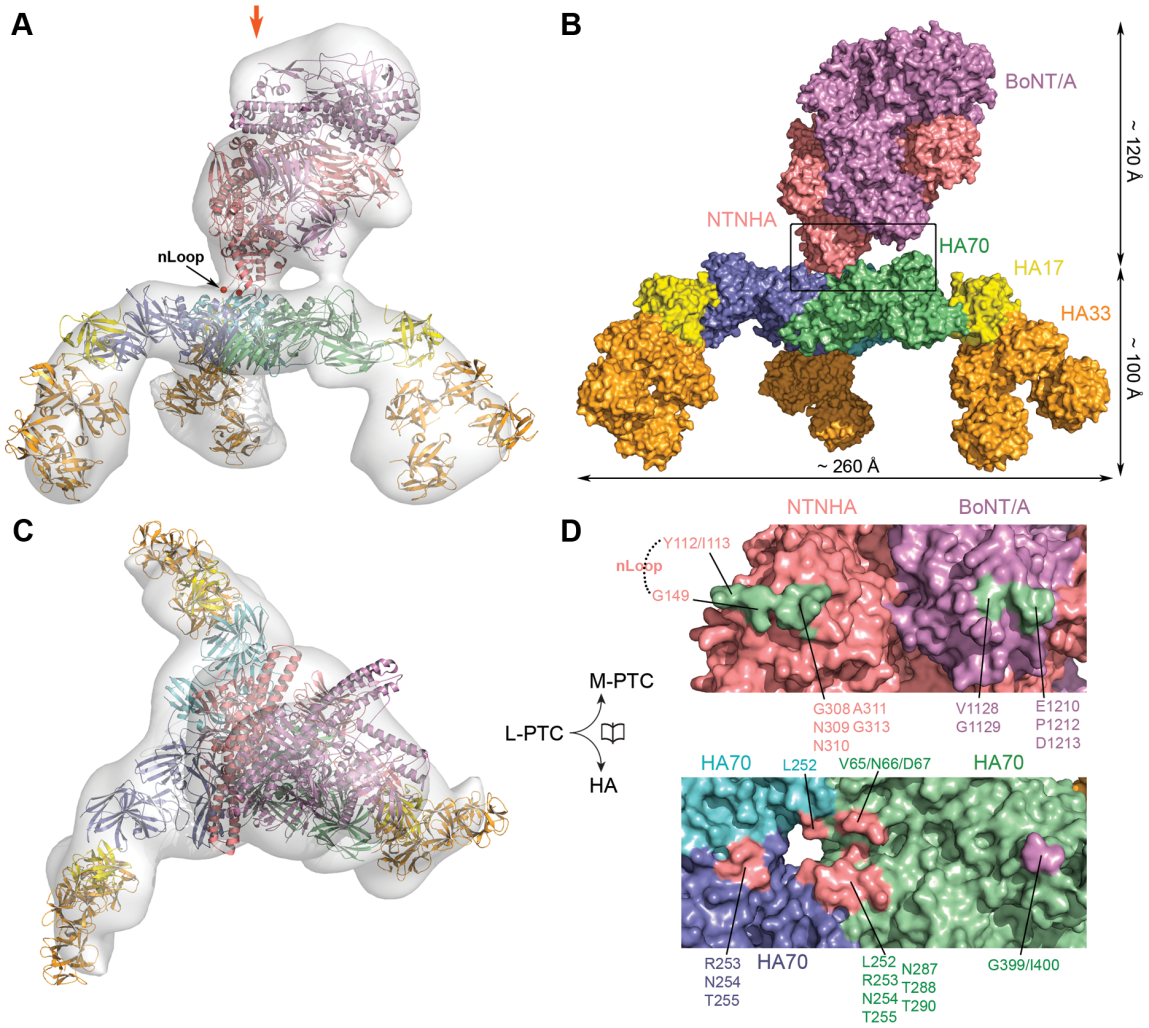
The molecular architecture of L-PTC/A. (A) 3D-EM reconstruction of L-PTC/A. Structural models of the M-PTC and the HA complex were fit into the EM envelope. The red arrow indicates the viewing direction of (C). (B) Surface representation of L-PTC/A in the same orientation as (A). (C) A different view of L-PTC/A. (D) An open-book view of the interface that is highlighted in the box in (B). See Fig. S2 in Text S1 for stereo versions of (A) and (B).

The high toxicity of BoNT/A prevents imaging of the fully active toxin by cryo-EM. So, we began our analysis with negative-staining EM and determined the 3D molecular envelope of L-PTC/A at ∼31 Å resolution (Fig. 1A–C and Fig. S1 in Text S1). The M-PTC moiety was clearly identified in the EM density map based on its crystal structure [5]. Beneath the M-PTC, the HAs adopt a symmetric three-blade architecture that is ∼100 Å tall and ∼260 Å wide between the tips of neighboring blades. Surprisingly, the M-PTC and the HA complex are relatively independent of each other and associate only through two small interfaces (Fig. 1D and Fig. S2 in Text S1). This arrangement contrasts with the extensive interactions between BoNT/A and NTNHA that are required for mutual protection in the GI tract [5], suggesting that the HA complex might play a minimal role in BoNT protection. We did not observe the LL-PTC under EM, which has been proposed to be a dimer of the L-PTC with a molecular weight of ∼900 kDa that might only occur at high concentrations [20], [30].

### *In vitro* reconstitution of the HA complex

To determine the molecular architecture of the HA complex, we produced highly homogeneous recombinant proteins of HA70, HA33, HA70–HA17, HA17–HA33, HA70^D3^(residues Pro378–Asn626)–HA17–HA33 (termed the mini-HA complex), and the complete HA70–HA17–HA33 (the HA complex). HA17 formed inclusion bodies and heterogeneous soluble aggregates when expressed and purified alone. This is probably due to the large hydrophobic patches on its surface, which are protected by its binding partners within the HA complex. We then systematically analyzed the solution association of these individual proteins and their complexes using analytical ultracentrifugation (AUC), which was performed at pH 2.3 and 7.6 to mimic the physiological conditions in the GI tract (Table S1 in Text S1). Our data indicate that the HA complex assembles at both pHs as a hetero-dodecamer consisting of HA70, HA17, and HA33 in a 3∶3∶6 ratio to yield a ∼470 kDa complex. Specifically, homo-trimeric HA70 forms the core of the complex with each C-terminal HA70^D3^ domain binding to one HA17, which in turn simultaneously coordinates two HA33s.

### Structure of the HA complex

**Figure 2:**
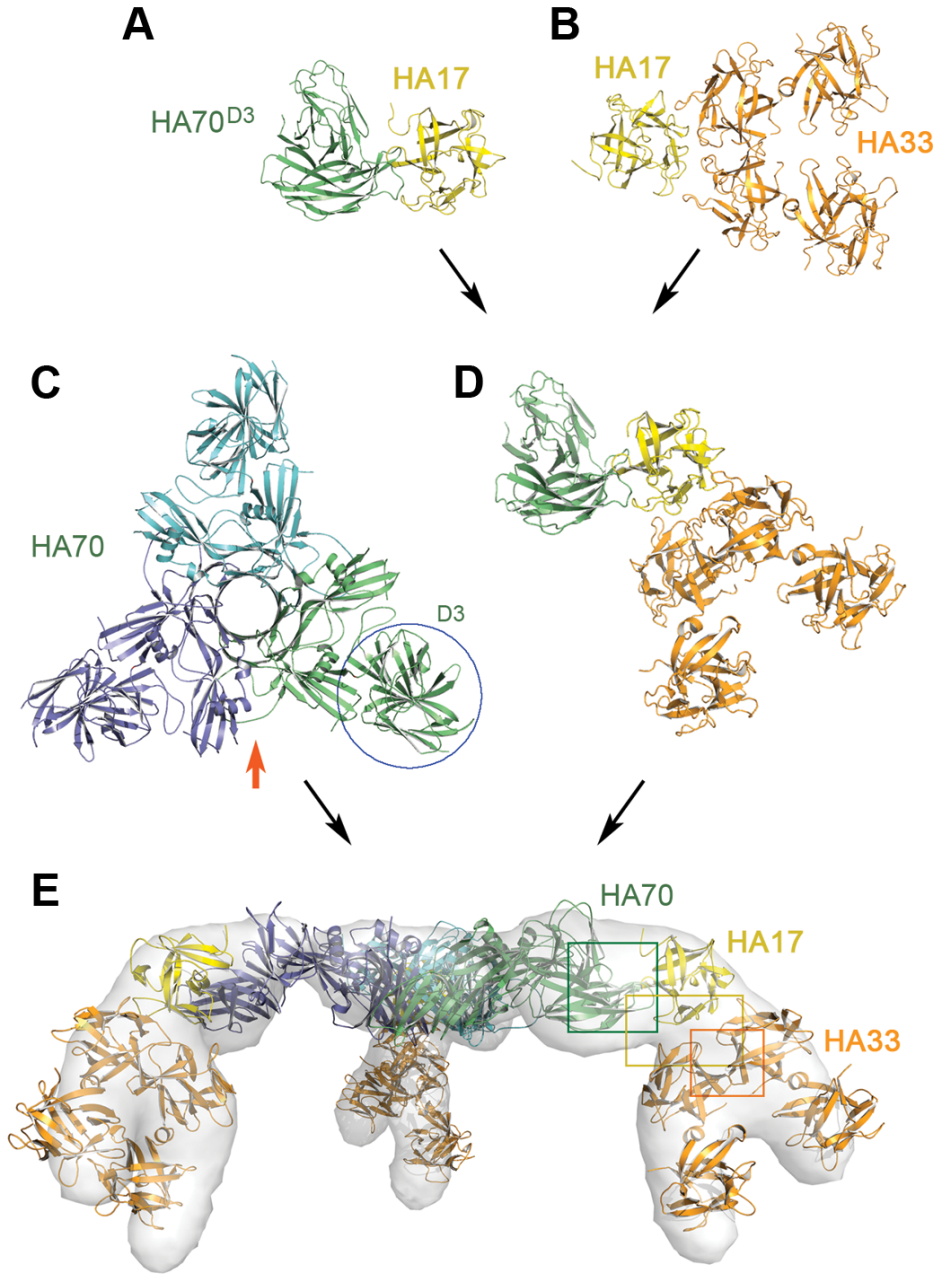
X-ray structures of the HA complex. (A) Structure of the HA70^D3^–HA17 complex at 2.4 Å resolution. (B) Structure of the HA17–HA33 complex at 2.1 Å resolution. (C) Structure of HA70 at 2.9 Å resolution. The red arrow indicates the viewing direction of (E). (D) Structure of the mini-HA complex (HA70^D3^–HA17–HA33) at 3.7 Å resolution. (E) 3D-EM reconstruction of the complete HA complex. The structure model of the HA complex was fit into the EM envelope. Open-book views of the interfaces highlighted in the green, orange, and yellow boxes are shown in Fig. 3.

We next separated the HA complex into two major components: the central hub composed of homo-trimeric HA70 and the blade composed of HA70^D3^–HA17–HA33. Their crystal structures were determined at 2.9 Å and 3.7 Å, respectively (Fig. 2C–D, Table S2 in Text S1, Fig. S3–S4 in Text S1). We also obtained a high-resolution structure of the blade by combining the structures of HA70^D3^–HA17 and HA17–HA33, which were determined at 2.4 Å and 2.1 Å, respectively (Fig. 2A–B and Fig. S5–S6 in Text S1). Each HA adopts an almost identical conformation in the independently solved structures, despite differences in crystal packing, suggesting that they represent physiologically relevant conformations.

HA70 consists of three domains (D1–3) (Fig. S3 in Text S1). The D1 and D2 domains, which adopt similar structures, mediate the trimerization of HA70 with each protomer contributing ∼3,100 Å^2^ of solvent-accessible area for interactions. The D3 domain, sitting at the tip of the trimer, is composed of two similar jelly-roll-like β-sandwich structures. The linker between D1 and D2 (residues Thr190–Ser205) is degraded and not visible in the crystal structure, which is reminiscent of the post-translational nicking of HA70 into ∼25 and ∼45 kDa fragments that occurs physiologically [20].

**Figure 3:**
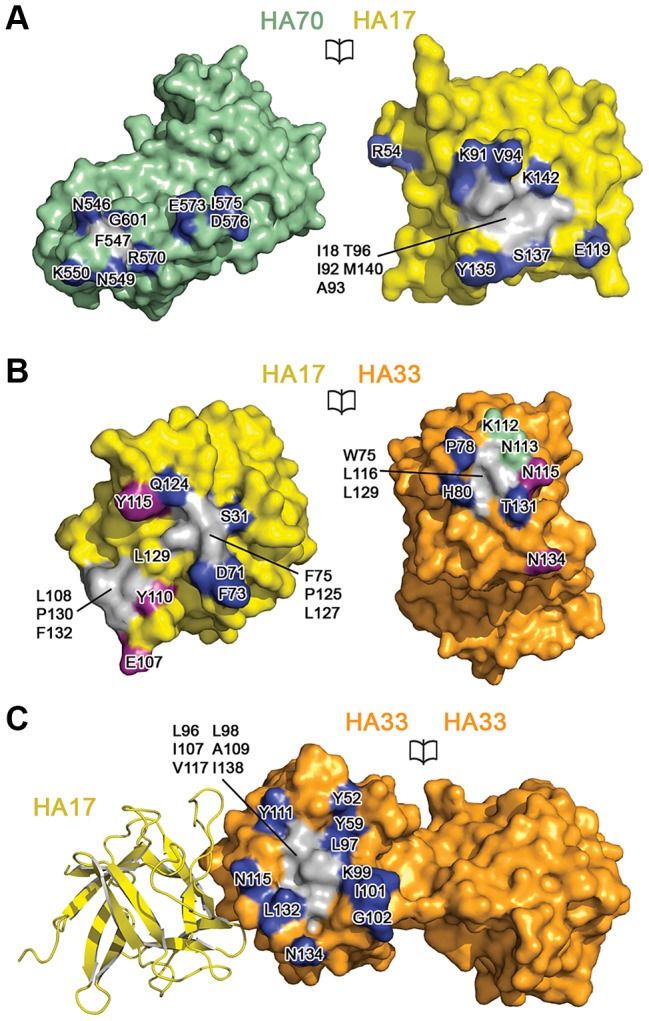
The 12-subunit HA complex is stabilized by extensive protein–protein interactions among three HAs. Interacting residues are labeled in open-book views of the interfaces. (A) Interface between HA70 and HA17. (B) Interfaces between HA17 and the two HA33s are indicated by purple and blue. The HA33 residues involved in both interfaces are in green. (C) Interface between two HA33s attached to the same HA17. See Fig. S5 and S6 in Text S1 for stereo versions of the detailed interactions.

HA17 has a compact β-trefoil fold and connects HA70 and HA33. Based on the crystal structure of the HA70^D3^–HA17 complex, the interactions between HA70 and HA17 bury a solvent-accessible area of ∼795 Å^2^ (per molecule) (Fig. 3A and Fig. S5 in Text S1). The structure of HA70^D3^ is almost identical to its equivalent domain in the full-length HA70 with a root-mean-square deviation (rmsd) of ∼0.93 Å over 232 Cα atoms. The major HA70–HA17 interactions are composed of 13 pairs of hydrogen bonds and salt bridges. In addition, HA70-Phe547 is buried in a hydrophobic region in HA17 composed of Ile18, Ile92, Ala93, Thr96, and Met140 (Fig. 3A and Fig. S5 in Text S1).

HA17 simultaneously binds to two HA33 molecules that form a dumbbell-like shape composed of two β-trefoil domains linked by an α-helix. The two pairs of HA17–HA33 interfaces bury a solvent-accessible area of ∼666 Å^2^ and ∼410 Å^2^ (per molecule), respectively (Fig. 3B and Fig. S6 in Text S1). The two HA33-binding interfaces on HA17 are adjacent but non-overlapping. HA17 contributes seven and four pairs of hydrogen bonds and salt bridges to bind the two HA33 molecules, respectively. Complementing these hydrophilic interactions, the two HA33s contain a hydrophobic surface (Trp75/Leu116/Leu129) that interacts with two neighboring hydrophobic patches on the HA17 surface (Phe75/Pro125/L127 and Leu108/Pro130/Phe132) (Fig. 3B).

The two molecules of HA33 in each blade of the HA complex are almost identical (rmsd of ∼0.35 Å over 286 Cα atoms) and bury a solvent-accessible area of ∼961 Å^2^ (per molecule) between them (Fig. 3C). All the interacting residues are in the N-terminal domain of HA33, whereas the interface consists of hydrophilic interactions on the periphery and a hydrophobic core in the center (Fig. S6C in Text S1). Due to the two-fold symmetry between the two molecules, intra-HA33 interactions are generally symmetric.

Finally, we assembled the subunit crystal structures to create a complete structure of the HA complex (Fig. 2E). The 12-subunit HA complex is stabilized by numerous protein–protein interactions that include interactions among the HA70s of the central trimer, between HA70 and HA17, between HA17 and the two HA33 molecules, and between the two HA33s in each blade. The assembled HA complex structure could be unambiguously docked into the 3D-EM density of the native L-PTC/A (correlation coefficient, CC∼87.7%) (Fig. 1), whereas a small difference was observed in the C-terminal domain of HA33 due to its structural flexibility. We also performed an independent 3D-EM reconstruction of our recombinant, *in vitro*-reconstituted HA complex at ∼31 Å resolution (CC∼93.1%) (Fig. 2E), and found it to be almost identical to the HA complex present in the L-PTC.

### The bimodular architecture of the L-PTC

The crystal structure of the M-PTC was unambiguously docked into the 3D-EM density of the native L-PTC (CC∼87.3%), which is situated on top of the HA complex, yielding a ∼760 kDa 14-subunit complex (Fig. 1 and Fig. S2 in Text S1). BoNT/A interacts with the HAs only through its receptor-binding domain (H_C_ domain).The interface is likely composed of Gly399 and Ile400 in HA70 and Val1128, Gly1129, Glu1210, Pro1212, and Asp1213 in H_C_ (pairwise Cα–Cα distance within 15 Å) (Fig. 1D and Fig. S7A in Text S1). Gly399 and Ile400 of HA70 are located in a loop that has weak electron density in the crystal structures, suggesting high flexibility. Moreover, the potentially interacting residues in H_C_ are located in two flexible loops and not conserved among various BoNT serotypes (Fig. S7B in Text S1). Thus, the BoNT/A–HA70 interface in the L-PTC may be formed by induced fit.

The major interface between the M-PTC and the HAs is mediated by NTNHA. The potential interface residues in NTNHA, which are within 12 Å Cα–Cα distance of the HAs, are located in loop Gly308–Gly313 and the residues flanking loop Gly116–Ala148 (nLoop) [5]. The corresponding interface residues in the HA complex are located around the center of the HA70 trimer (Fig. 1D). The nLoop displays no visible electron density in the structure of the M-PTC and is spontaneously nicked in the free NTNHA or the M-PTC [5], [31], [32], [33], [34]. However, the nLoop remains intact in the L-PTC, suggesting it may be buried by the HA complex [30], [31], [35]. We found that the synthetic nLoop peptide binds to HA70 with high affinity (*K*_d_∼340 nM) at a stoichiometry of one nLoop to one HA70 trimer (Table S3 in Text S1). This finding unambiguously established the orientation of the pseudo 2-fold symmetric M-PTC on top of the HA complex. The nLoop of NTNHA binds strongly to HA70 at pH 7.6, which is in contrast to the M-PTC that dissembles and releases BoNT/A at neutral or basic pH [5], [30]. This suggests that BoNT may be the only component released from the L-PTC in response to the pH change encountered upon entering the circulation [30].

### Only the complete HA complex compromises the integrity of the epithelial cell layer

The HA complex and the M-PTC are stable at low pH (e.g., pH 2.3) and are resistant to digestive proteases, as shown by *in vitro* cleavage by trypsin and pepsin (Fig. S8 in Text S1) [5]. The loose association between these two complexes suggests that they may have distinct functions during oral intoxication. The penetration of BoNT through an epithelial cell barrier to reach the general circulation is the first essential step of oral BoNT intoxication, which prompted us to investigate the role of HAs in BoNT/A absorption from the GI tract. For this study, we used the well-characterized intestinal epithelial cell line Caco-2. Although derived from a human colon adenocarcinoma, Caco-2 cells differentiate to form a polarized epithelial cell monolayer that provides a physical and biochemical barrier to the passage of ions and small molecules, resembling the uptake and barrier properties of the small intestinal epithelial layer [36], [37], [38], [39]. Caco-2 cells have been extensively used to investigate their permeability upon infection, e.g. by rotavirus [40] or enteropathogenic *E. coli* [41], and transcytosis upon intoxication with cholera toxin [42] or BoNT [43], [44], [45]. Furthermore, it was demonstrated that the transepithelial electrical resistance (TER) of Caco-2 cell monolayers was reduced by the L-PTC of BoNT/A and B. Although the mechanism by which this may occur is unclear, BoNT absorption has been proposed to occur via the paracellular route [27], [28], [29].

**Figure 4:**
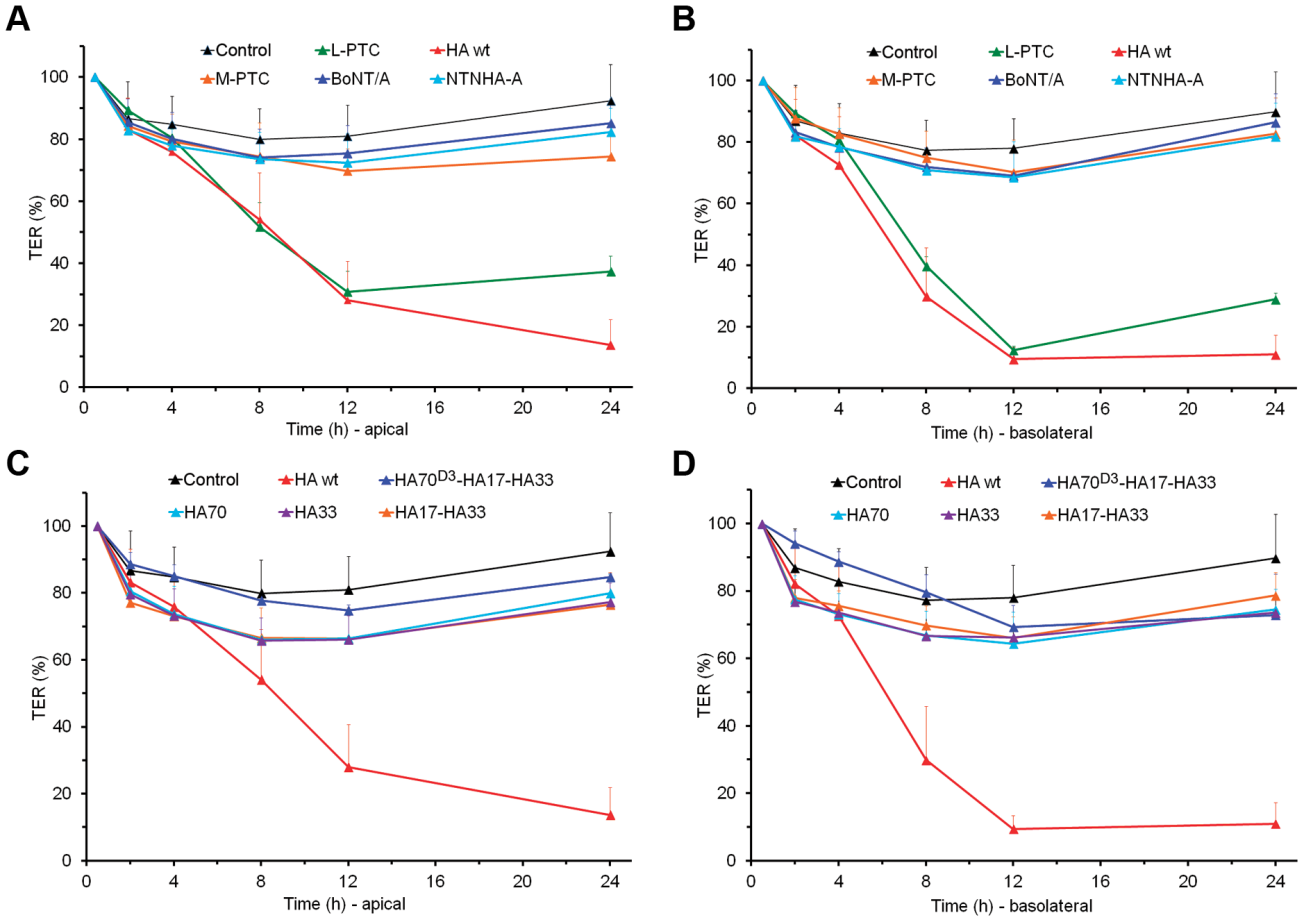
The fully assembled HA complex markedly reduced the TER of human intestinal Caco-2 cell monolayers. Caco-2 cells were grown on transwell filter membranes into confluent polarized monolayers. (A, B) TER was measured following application of the L-PTC, the HA complex, the M-PTC, BoNT/A, or NTNHA-A to the apical (A; 58 nM) or basolateral (B; 17 nM) chambers. (C, D) TER was measured when the HA complex (HA wt), the mini-HA complex (HA70^D3^–HA17–HA33), HA70 trimer, HA33, or the HA17–HA33 complex were applied to the apical (C; 58 nM) or basolateral (D; 17 nM) chambers. Values are means ± SD (*n* = 4–12).

We found that treatment of Caco-2 cells with the *in vitro*-reconstituted HA complex markedly reduced the TER. This effect was more marked when the HA complex was applied to the cell monolayer from the basolateral side than from the apical side, which needed ∼17 nM and ∼58 nM to achieve a 90% and 70% decrease in TER after 12 hours, respectively (Fig. S9A–B in Text S1). Remarkably, the potency of the isolated HA complex was similar to that of the intact L-PTC (Fig. 4A–B). In contrast, there was no effect on Caco-2 TER by BoNT/A, NTNHA, the M-PTC, or by the subunits of the HA complex, including HA70, HA33, the HA17–HA33 complex, and the mini-HA complex (Fig. 4C–D). Taken together, these data suggest that the fully assembled HA complex is the functional unit of the L-PTC that facilitates intestinal absorption of BoNT, and acts by compromising the integrity of the epithelial cell layer.

### The HA complex interacts with carbohydrates

Many human receptors for microbial pathogens or toxins are glycoconjugates. The L-PTC is known to initiate toxin absorption by binding to intestinal cell surface glycans [24], [25], [26]. We therefore performed a comprehensive thermodynamic analysis to characterize the interactions between HAs and several common monosaccharides and oligosaccharides (Fig. S10 and Table S3 in Text S1). We found that HA33 bound to lactose (Lac), N-acetyllactosamine (LacNAc), and galactose (Gal) with dissociation constants (*K*_d_) of ∼1.0 mM, ∼1.0 mM, and ∼1.8 mM, respectively, and that it also bound to isopropyl β-D-1-thiogalactopyranoside (IPTG) [46], a non-metabolizable galactose analog, with a *K*_d_ of ∼0.8 mM. HA70 bound to α2,3- and α2,6-sialyllactose (SiaLac), both with *K*_d_ of ∼0.5 mM, and displayed a lower affinity for N-acetylneuraminic acid (Neu5Ac) (*K*_d_∼7.8 mM). There was no overlap between the carbohydrate selectivity of HA70 and HA33.

**Figure 5:**
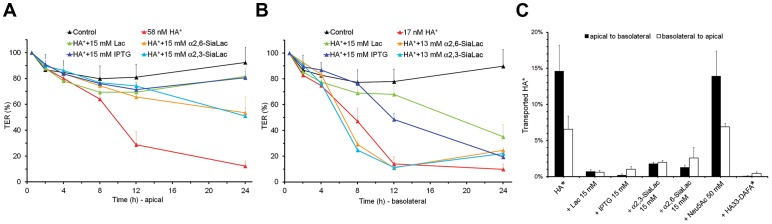
The HA complex interacts with carbohydrate receptors to cross epithelial cell monolayers. (A, B) TER of Caco-2 monolayers was measured when Alexa-488-labeled HA complex (HA*) pre-incubated with Lac, IPTG, α2,3-SiaLac, or α2,6-SiaLac was applied to the apical (A; 58 nM) or basolateral (B; 17 nM) chambers. Values are means ± SD (*n* = 4–12). (C) HA* (with or without carbohydrates) or the Alexa-488-labeled HA^33-DAFA^ complex (HA^33-DAFA^ *) was applied to the apical (at 58 nM) or basolateral (at 17 nM) chamber. The fluorescence signals in both chambers were quantified after 24 hours and the amount of transported HA*/HA^33-DAFA^ * was expressed as a percentage of the total HA*/HA^33-DAFA^ * used. Values are means ± SD (*n* = 3–22).

To determine the physiological relevance of these HA–glycan interactions during toxin absorption, we examined their ability to interfere with the HA complex-mediated disruption of Caco-2 TER. Lac, Gal, and IPTG markedly inhibited the TER reduction induced by the HA complex, and showed higher potencies when applied to the apical than to the basolateral compartment (Fig. 5A–B and Fig. S11A–D in Text S1). In contrast, α2,3- and α2,6-SiaLac, and to a lesser extent Neu5Ac, inhibited the decrease in TER only when applied apically, albeit more weakly than Lac (Fig. 5A–B and Fig. S11E–F in Text S1). We then examined the transport of the HA complex across the Caco-2 monolayer using a fluorescence-labeled HA complex (HA*) (Fig. 5C). Lac and IPTG efficiently inhibited the transport of HA* when it was applied to the apical or basolateral chamber. Blocking the transport of HA* via α2,3- and α2,6-SiaLac was more potent toward the basolateral compartment than toward the apical side. Neu5Ac at 50 mM did not inhibit transport of HA* from either side of the Caco-2 cell monolayer. These data are consistent with the ability of these carbohydrates to inhibit TER reduction induced by the HA complex. Collectively, these results suggest that the binding of HAs to Neu5Ac- and Gal-containing glycans on epithelial cells is essential for the transport of BoNT across the intestinal wall. Moreover, the carbohydrate receptors may play a more important role in the initial L-PTC absorption in the intestinal lumen, whereas other host receptors (e.g., E-cadherin) are involved once it gains access to the basolateral side.

### The HA complex recognizes its carbohydrate receptors with high specificity

**Figure 6:**
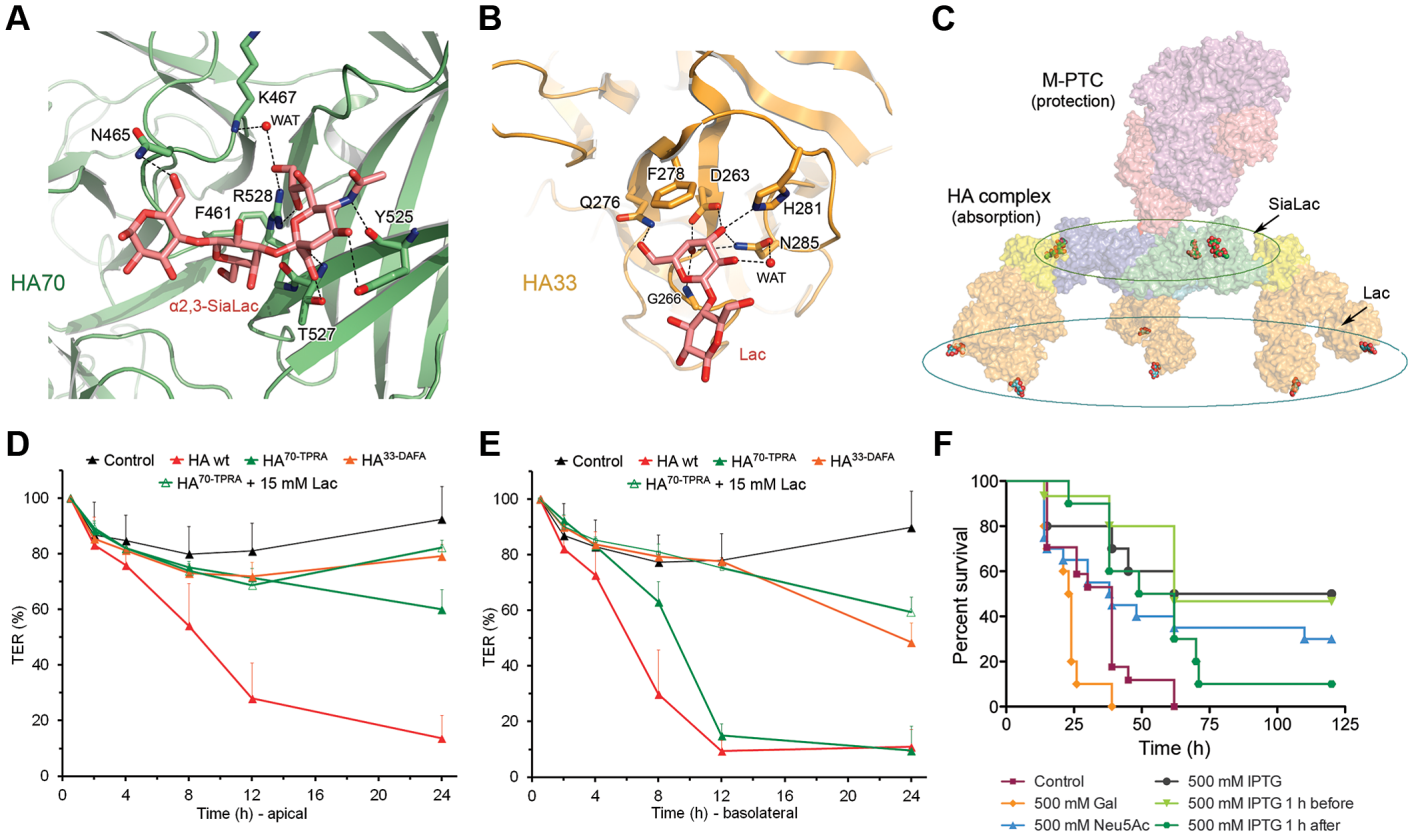
The HA complex mediates BoNT absorption through multivalent interactions with glycan receptors. Close-up views of HA70–α2,3-SiaLac and HA33–Lac interactions are shown in (A) and (B), respectively. Key HA residues involved in glycan coordination are shown as sticks. Hydrogen bonds are indicated by black dashed lines. (C) The HA complex has nine glycan-binding sites. (D, E) TER of the Caco-2 monolayers were measured after application of the wild-type HA complex (HA wt), the HA^70-TPRA^ complex, or the HA^33-DAFA^ complex to the apical (D) or basolateral (E) chambers. Values are means ± SD (*n* = 4–12). (F) Survival comparisons of mice treated orally with L-PTC/A in the presence or absence of IPTG, Neu5Ac, and Gal.

To fully understand the binding specificity, we determined the crystal structures of HA70 in a complex with α2,3- or α2,6-SiaLac (Fig. 6 and Table S4 in Text S1). We found that α2,3- and α2,6-SiaLac bound to the same region in the D3 domain of HA70, where the terminal Neu5Ac in both glycans mediates the majority of the HA70–glycan interactions through six pairs of hydrogen bonds (Fig. 6A and Fig. S12 in Text S1). Mutating the Neu5Ac-binding residues (e.g. T527P, R528A, or T527P/R528A) completely abolished the binding (Table S3 in Text S1). The Neu5Ac-binding mode in HA70-A is also conserved in HA70-C (Fig. S13 in Text S1) [15], [16], suggesting HA70 is unlikely to be a major determinant of the host tropism of various BoNT serotypes.

In contrast to the well-defined conformation of Neu5Ac, the Gal–Glc moiety seems to have a larger structural flexibility and is not essential to HA70–glycan recognition. Specifically, α2,3-SiaLac adopts a linear conformation, which is likely stabilized by a Glc-mediated crystal contact with its symmetry mate. However, α2,6-SiaLac binding to the same site adopts a folded conformation in which there is no crystal contact and Glc has no visible electron density (Fig. S12B in Text S1). Furthermore, these conformations are also different than that observed in the structures of α2,3- and α2,6-SiaLac when they bound to HA70-C, despite the conserved Neu5Ac-binding mode [16]. The different glycan conformations and the weak electron densities for Gal–Glc observed here are probably due to the intrinsic flexibility of SiaLac in solution [47]. The ability of HA70 to bind SiaLac with different glycosidic linkages contrasts with the binding profile of influenza virus HA. Neu5Ac binds to a deep pocket in influenza HA, which restricts the composition and topology of glycans that can bind to influenza HA [48], [49], [50]. In contrast, the Neu5Ac-binding site in HA70 is on a flat surface, allowing more freedom for additional glycan binding beyond the terminal Neu5Ac.

We also determined the crystal structures of the HA17–HA33 complex bound with Gal, Lac, or LacNAc (Table S4 in Text S1). All three bind to an identical site in HA33, where the HA33–glycan interactions are mediated only by the Gal moiety through extensive hydrogen bonding and a crucial stacking interaction between Phe278 and the hexose ring of Gal (Fig. 6B). The HA33–Gal interaction is well-defined and identical for the two HA33 molecules in one asymmetric unit (AU). The Glc or GlcNAc moiety does not directly interact with HA33. One Glc/GlcNAc in the AU is involved in a crystal packing and shows clear electron density, while the density for the other copy is weakly defined; the latter is likely caused by the weak HA33–glycan binding affinity and intrinsic structural flexibility of HA33 that will be discussed later (Fig. S12D–F in Text S1). To further confirm the structural findings, we mutated the Gal-binding residues in HA33 (e.g., D263A or F278A) and found that these mutations almost completely abrogated the Lac binding (Table S3 in Text S1).

Gal binds at an equivalent site in HA33 of L-PTC/C (HA33-C) (Fig. S14 in Text S1) but with ∼15-fold lower binding affinity than with HA33-A [13], which is likely caused by the replacement of Phe278 in HA33-A with Asp271 in HA33-C. In addition, HA33-C binds Neu5Ac in an adjacent binding site [51]. However, HA33-A does not bind Neu5Ac-containing sugars because the key Neu5Ac-binding residues in HA33-C, Trp176 and Arg183, are replaced in HA33-A with Tyr180 and Asn187, respectively (Table S3 in Text S1). These differences between HA33-A and HA33-C indicate that the known host susceptibility to different BoNT serotypes may be determined in part by the interaction between HA33 and host glycan receptors.

To further analyze the functional role of BoNT's glycan receptors, we “knocked-down” specific glycan binding to the HA complex using structure-based mutagenesis. The HA^33-DAFA^ complex (composed of the wild-type (WT)-HA70, WT-HA17, and HA33-D263A/F278A) did not bind to Gal, whereas the HA^70-TPRA^ complex (composed of the HA70-T527P/R528A, WT-HA17, and WT-HA33) failed to bind to Neu5Ac (Table S3 in Text S1). We found that the HA^33-DAFA^ complex did not reduce TER when applied from either side of the Caco-2 cell monolayer. Furthermore, the loss of the Gal-binding site prevented the transport of HA^33-DAFA^ through the Caco-2 monolayer (Fig. 5C), indicating the crucial role of the carbohydrate interaction during transcytosis. The HA^70-TPRA^ complex maintained activity only when applied from the basolateral side, which was inhibited by Lac (Fig. 6D–E). These data suggest that there are at least two steps at which HA–glycan interactions play an important role in toxin absorption. Both Neu5Ac- and Gal-containing glycans are important for the initial L-PTC absorption in the intestinal lumen, but Gal-containing receptors on the basolateral surface of the epithelial cells may also participate, presumably in facilitating transport via the paracellular route [27], [28], [29].

### Carbohydrate receptor mimics could inhibit BoNT/A oral toxicity

To determine whether the glycans could interfere with BoNT absorption *in vivo*, we examined the effect of the monosaccharides Neu5Ac, Gal, and IPTG on the oral toxicity of L-PTC/A in mice [4]. Concomitant oral administration of L-PTC/A and IPTG at 500 mM significantly extended the median survival time (MST) of animals to ∼91 hours compared with ∼39 hours for the control group. Furthermore, IPTG was effective when it was administered one hour prior to treatment with L-PTC/A with an increase of MST to ∼62 hours. Some improvement in survival was also evident with IPTG treatment one hour after intoxication with L-PTC/A, with an increase of MST to ∼55 hours (Fig. 6F). Since IPTG does not affect the neurotoxicity of BoNT/A based on the mice phrenic nerve hemidiaphragm assay, this finding suggests that receptor mimics could block BoNT/A intestinal absorption at an early point of oral intoxication. Gal and Neu5Ac (up to ∼500 mM) did not confer significant protection, most likely due to their low binding affinity and/or metabolism (Fig. S15 in Text S1).

## Discussion

Here, we report the complete structure of a 14-subunit ∼760 kDa L-PTC/A, which is achieved by building novel crystal structures of each subunit into 3D-EM reconstruction. To our knowledge, this is the largest bacterial toxin complex known to date. The L-PTC/A adopts a unique bimodular architecture, whereas BoNT/A and NTNHA form a compact M-PTC and three HA proteins adopt an extended three-arm shape. Our results conclude the same stoichiometry and a similar overall architecture as suggested by recent EM studies of L-PTC/A, B, and D [14], [22]. Furthermore, our complementary crystallographic, EM, and biochemical studies have revealed for the first time that both BoNT/A and NTNHA are involved in interactions with the HA complex, and that the two modules associate through two small interfaces, in contrast to numerous protein–protein interactions within each module.

Aside from a small interface involving the BoNT/A receptor-binding domain, the majority of the interactions between the M-PTC and the HAs are mediated by the NTNHA nLoop. In spite of the overall structural similarity between BoNT/A and NTNHA, the nLoop is a unique feature of NTNHA, which is fully exposed on the M-PTC surface [5]. The nLoop is conserved in the NTNHAs that shield BoNT/A1, B, C, D, and G, and assemble with HAs into the L-PTC. However, the nLoop is missing in NTNHAs that assemble with BoNT/A2, E, and F, which do not have accompanying HA proteins and only form the HA-negative M-PTC [11], [52], [53]. We have found that one molecule of the synthetic nLoop peptide binds to the trimeric HA70 with a high affinity, clearly suggesting that the nLoop is bridging the M-PTC and the HA complex. This is consistent with previous reports that the nLoop is intact in the context of the L-PTC but spontaneously nicked in the free NTNHA or the M-PTC [5], [30], [31], [32], [33], [34].

Structural and sequence analyses suggest that the 12-subunit architecture of the HA complex is likely conserved across different BoNT serotypes [14], [22]. For example, pairwise structural comparisons yield rmsd of ∼1.28 Å (582 Cα atoms) and ∼1.20 Å (137 Cα atoms) for HA70-A/HA70-C and HA17-A/HA17-D, respectively; they are ∼0.87 Å (129 Cα atoms) and ∼1.23 Å (134 Cα atoms) for the two domains of HA33 between serotypes A and D and similarly between HA33-A and HA33-C [13], [14], [15], [16]. Moreover, the protein–protein interactions within the HA70 trimer and between HA17 and HA33 are largely conserved among our crystal structures of serotype A and the known crystal structures of serotypes C and D.

Despite the largely rigid structure of the HA complex, HA33 seems to have an intrinsic structural flexibility. The N-terminal domain of HA33 is fixed in the HA complex through extensive inter-HA33 and HA17–HA33 interactions, but its C-terminal domain is largely unrestricted. When comparing two HA33-A structures that were determined in different crystal forms, we found that the N- and C-terminal domains of HA33-A twist against each other by ∼14° (Fig. S16 in Text S1) [17]. A more significant conformational change is observed between HA33-A and C (∼61°) and HA33-A and D (∼65°) (Fig. S16 in Text S1) [13], [14]. In the context of the assembled HA complex, such a conformational change leads to a shift up to ∼23 Å for the C-terminal Gal-binding site in HA33. We suggest that HA33 could require such structural flexibility to achieve its multivalent host-receptor binding in the intestine.

The loose linkage between the M-PTC and the HA complex clearly suggests divided functions. We previously reported that the M-PTC's compact structure protects BoNT against digestive enzymes and the extreme acidic environment of the GI tract [5], [23]. We now show that the HA complex is mainly responsible for BoNT absorption in the small intestine, through binding to specific host carbohydrate receptors. This new finding permitted the identification of IPTG as a prototypical oral inhibitor that extends survival following lethal oral BoNT/A intoxication of mice. Multivalent interactions involving nine binding sites for Neu5Ac- and Gal-containing glycans increase the overall avidity of binding between the L-PTC and glycans on the epithelial cell surface, and thus compensate for the modest glycan-binding affinities at individual binding sites (Fig. 6C). Similarly, the potency of carbohydrate receptor mimics could be improved by optimizing the HA–glycan interactions as revealed here or by introducing new HA–inhibitor interactions at individual binding sites based on rational design, as well as by designing multivalent inhibitors. Although such inhibitors cannot be used to treat fully developed food-borne botulism, they could provide temporary protection upon pre-treatment and could also be useful for cases of intestinal colonization with *C. botulinum* spores such as in cases of infant or adult intestinal botulism. Our results also suggest that the L-PTC could be exploited for alternative applications. For example, protein-based therapeutics could be coupled to the modified non-toxic L-PTC to allow oral delivery by improving drug stability, absorption efficiency, and bioavailability.

## Materials and Methods

### Ethics statement

The Institutional Animal Care and Use Committee of the United States Department of Agriculture, Western Regional Research Center approved the experimental and husbandry procedures used in these studies (protocol # 12-2). All animal experiments were conducted under the guidelines of the U.S. Government Principles for the Utilization and Care of Vertebrate Animals Used in Testing, Research and Training.

### Construct design and cloning

The sequences corresponding to full-length HA70 (residues M1–N626), HA70^D3^ (residues P378–N626), full-length HA17 (residues M1–I146), and full-length HA33 (residues M1–P293) from BoNT/A1-producing *C. botulinum* strain 62A were cloned into expression vectors pQE30, pGEX-6p-1, pRSFDuet-1, and pET28a, respectively. In addition, HA17 and HA33 were cloned into the bicistronic pRSFDuet-1 for co-expression.

To facilitate protein purification, a 6×His tag followed by a thrombin cleavage site was introduced to the N-termini of HA70, HA17, and HA33. HA70^D3^ was cloned into pGEX-6p-1 following the N-terminal GST and a PreScission cleavage site. For HA17 and HA33 in the pRSFDuet-1 vector, HA17 was produced with an N-terminal 6×His tag followed by a PreScission cleavage site, while HA33 had no affinity tag. All HA33 or HA70 mutations were generated by QuikChange site-directed mutagenesis (Stratagene).

### Protein expression and purification

Four different protein expression schemes were used to produce the individual HAs or HA complexes. (1) HA70 (pQE30), HA70^D3^ (pGEX-6p-1), and HA33 (pET28a) were expressed alone; (2) HA70 (pQE30) and HA17 (pRSFDuet-1) were co-transformed into bacteria and co-expressed; (3) HA70^D3^ (pGEX-6p-1) and HA17 (pRSFDuet-1) were co-transformed into bacteria and co-expressed; and (4) HA17 and HA33 were co-expressed using the bicistronic pRSFDuet-1 vector.

All recombinant proteins were expressed in the *E. coli* strain BL21-RIL (DE3) (Novagen). Bacteria were grown at 37°C in LB medium in the presence of the appropriate selecting antibiotics. Expression was induced with 1 mM isopropyl-β-D-thiogalactopyranoside (IPTG) when OD_600_ had reached 0.7. The temperature was then decreased to 18°C and expression was continued for ∼16 hours. The cells were harvested by centrifugation and stored at −20°C until use.

For purification of His-tagged proteins (HA70, HA33, the HA70–HA17 complex, and the HA17–HA33 complex), proteins were bound to a Ni-NTA (nitrilotriacetic acid, Qiagen) affinity column in a buffer containing 50 mM Tris (pH 8.0) and 400 mM NaCl, and subsequently eluted in the same buffer containing 300 mM imidazole. The eluted fractions of each protein were pooled and dialyzed overnight at 4°C against a buffer composed of 20 mM Tris (pH 8.0) and 50 mM NaCl, then the His-tag was removed with thrombin (for HA70, HA33, and the HA70–HA17 complex) or PreScission protease (for the HA17–HA33 complex). GST-tagged HA70^D3^ and the HA70^D3^–HA17 complex were purified using Glutathione Sepharose 4B resins (GE Healthcare) in phosphate-buffered saline, and eluted from the resins after on-column cleavage using PreScission protease.

The following three schemes were used to further purify the proteins. (1) HA70 and the HA70–HA17 complex was purified by MonoQ ion-exchange chromatography (GE Healthcare) in a buffer containing 20 mM Tris (pH 8.0) and eluted with a NaCl gradient, followed by Superdex 200 size-exclusion chromatography (GE Healthcare) in 20 mM Tris (pH 8.0) and 50 mM NaCl. (2) HA33 and the HA17–HA33 complex were purified by MonoS ion-exchange chromatography in a buffer containing 20 mM sodium acetate (pH 5.0) and eluted with a NaCl gradient, followed by Superdex 200 chromatography in 20 mM Tris (pH 8.0) and 50 mM NaCl. (3) HA70^D3^ and the HA70^D3^–HA17 complex were purified by MonoQ ion-exchange chromatography in 20 mM Tris (pH 8.0) followed by Superdex 200 chromatography in 20 mM Tris (pH 8.0) and 50 mM NaCl for HA70^D3^ or 20 mM sodium citrate (pH 5.0) and 100 mM NaCl for the HA70^D3^–HA17 complex. Each protein or protein complex was concentrated to ∼3–6 mg/ml using Amicon Ultra centrifugal filters (Millipore) and stored at −80°C until used for further characterization or crystallization.

The purified HA70 was labeled with Alexa Fluor® 488 carboxylic acid, succinimidyl ester (Life Technologies) according to the manufacturer's instructions. The labeled HA70 was further purified by Superdex 200 chromatography in 20 mM Tris (pH 8.0) and 50 mM NaCl. The calculated dye to protein ratio was ∼2 moles of dye per mole of monomeric HA70.

### *In vitro* reconstitution of the HA complex

The HA17–HA33, the HA70–HA17, and the HA70^D3^–HA17 complexes were produced by co-expression and co-purification as described above. To assemble the mini-HA complex (HA70^D3^–HA17–HA33), the purified HA33 and the HA70^D3^–HA17 complex were mixed at a molar ratio of ∼2.5∶1 and incubated at 4°C overnight. The excess HA33 was removed by Superdex 200 chromatography with 20 mM Tris (pH 7.6) and 50 mM NaCl. The fully assembled HA complex was reconstituted by mixing the purified HA70 and the HA17–HA33 complex at a molar ratio of ∼1∶1.3. The mixture was incubated at 4°C overnight and the excess HA17–HA33 complex was removed from the mature HA complex by Superdex 200 chromatography with 20 mM Tris (pH 7.6) and 50 mM NaCl. The fluorescence-labeled HA complex was prepared with Alexa Fluor® 488-labeled HA70 and unlabeled HA17–HA33 complex (HA*) or HA17–HA33^DAFA^ complex (HA33^DAFA^*) using a similar protocol.

### Analytical ultracentrifugation (AUC)

Sedimentation equilibrium (SE) experiments were performed in a ProteomeLab XL-I (BeckmanCoulter) analytical ultracentrifuge. Purified HA samples were dialyzed extensively against a buffer containing 50 mM Tris (pH 7.6) and various NaCl concentrations, or 50 mM citric acid (pH 2.3) and various NaCl concentrations. Protein samples at concentrations of 0.4, 0.2, and 0.1 unit of OD_280_ were loaded in 6-channel equilibrium cells and centrifuged at 20°C in an An-50 Ti 8-place rotor at the first speed indicated until equilibrium was achieved and thereafter at the second speed. HA33 was analyzed at rotor speeds of 19,000 and 22,000 rpm. The HA17–HA33 and the HA70^D3^–HA17–HA33 complexes were analyzed at 12,000 and 14,000 rpm. The HA70–HA17 and the HA70–HA17–HA33 complexes were run at speeds of 6,000 and 8,000 rpm. For each sample, data sets for the two different speeds were analyzed independently using HeteroAnalysis software (by J.L. Cole and J.W. Lary, University of Connecticut). Three independent experiments were performed for each sample.

The AUC data showed that HA33 is predominantly monomeric in solution at pH 2.3 or pH 7.6. HA17–HA33 forms a tight complex at pH 2.3 or pH 7.6, and the data were best fit to a model composed of one HA17 and two HA33 molecules. The HA70–HA17 complex precipitated at pH 2.3 and was therefore analyzed only at pH 7.6. The best fits for HA70–HA17 clearly suggested a complex composed of three HA70 and three HA17 molecules. The data for the HA70^D3^–HA17–HA33 complex were best fit to a model composed of one HA70^D3^, one HA17, and two HA33 molecules. HA70–HA17–HA33 forms a tight complex containing three HA70, three HA17, and six HA33. Weak dimerization was observed for the mini-HA complex (*K*_d_ of ∼23.1 µM) and the full HA complex (*K*_d_ of ∼10.9 µM) at pH 7.6 in the presence of 100 mM NaCl, but was not observed at higher ionic strength. The weak oligomerization *K*_d_ suggests that the mini-HA and the full HA complex are monomeric under physiological conditions.

### 3D-EM of the L-PTC and the HA complex

The L-PTC of BoNT/A was obtained from List Biological Laboratories, Inc. (Campbell, California) and Miprolab GmbH (Göttingen, Germany). The recombinant HA complex was reconstituted *in vitro* as described above. Negatively stained EM specimens were prepared following a previously described protocol [54]. Briefly, 3 µl of the L-PTC (∼0.02 mg/ml in 20 mM MES, pH 6.2, and 100 mM NaCl) or the HA complex (∼0.01 mg/ml in 20 mM Tris, pH 7.6, and 50 mM NaCl) was placed on a freshly glow-discharged carbon-coated EM grid, blotted with filter paper after 40 seconds, washed with two drops of deionized water, and then stained with two drops of freshly prepared 1% uranyl formate, which also served to fix the proteins.

Particle images were acquired using a 4k×4k TVIPS CCD camera on a Tecnai F20 electron microscope (FEI) equipped with a field emission electron source operated at 200 kV, at a nominal magnification of ∼70,000, resulting in a calibrated pixel size of 4.28 Å/pixel on the object scale after binning. The defocus values were set in the range of 1.5–3.2 µm. The electron dosage was ∼40 electrons/Å^2^. Image quality was monitored on the basis of power spectra quality. Particle boxing, CTF correction, initial model generation, 3D refinement, and resolution assessment were all carried out with the EMAN2 package [55]. Particles were semi-automatically boxed out and subjected to reference-free class-averaging using EMAN2. The standard EMAN2 initial model generation program (e2initialmodel.py) was used to obtain initial templates for refinement. With the use of this methodology, models were constructed from a series of randomly generated Gaussian blobs and refined against reference-free-generated 2D class averages. The resulting models were ranked on the basis of the agreement of the projection with the class average. The top initial templates were used as starting models for the subsequent refinement with EMAN2. For the L-PTC, no symmetry was imposed throughout the 3D reconstruction and refinement, while for the HA complex, a C3 symmetry was imposed. Refinement was terminated when no significant changes could be visually detected. A data set of 15,140 particles was used for the final reconstructed map of the L-PTC, for which the resolution was estimated to be ∼30.8 Å based on the resolution criteria of Fourier shell correlation (FSC) at 0.5 cutoff. A data set of 3,746 particles was used for the final reconstructed map of the HA complex, for which the resolution was estimated to be ∼30.6 Å. The density maps were filtered to 30 Å with the low-pass filter in EMAN2. Handedness of the maps was determined on the basis of the HA complex structure derived from crystal structures.

### Visualization and molecular docking

Visualization and rigid-body docking of atomic models into the 3D-EM density maps were performed using UCSF Chimera [56]. The Chimera Fit in Map utility, which maximizes the cross-correlation coefficient between the 3D-EM density map and the calculated density map (filtered to 30 Å) of the atomic structures, was used to optimize the docking of atomic structures into 3D-EM maps. After fitting refinement, the positions with highest correlation coefficient (cc) values were chosen. For the HA complex, the atomic structure derived from crystal structures of HA70 and the HA70^D3^–HA17–HA33 complex fitted the 3D-EM map very well (cc = 93.1%). There was a slight deviation at the C-terminal domain of HA33 that is located at the tip of the complex, which may be due to the structural flexibility of HA33. For the L-PTC, the densities for the M-PTC and the HA complex could be clearly identified in the 3D-EM reconstruction map, and the density for the HA complex was manually 3-fold averaged using Chimera and EMAN2. Atomic structures of the M-PTC and the HA complex were then docked separately into their 3D-EM densities, with highest cc values of 87.3% and 87.7%, respectively. The docked M-PTC and the HA complex were then merged to generate the complete pseudo-atomic model for the L-PTC.

### Crystallization

Initial crystallization screens were performed using a Phoenix crystallization robot (Art Robbins Instruments) and high-throughput crystallization screen kits (Hampton Research, Qiagen, or Emerald BioSystems), followed by extensive manual optimization. The best single crystals were grown at 18°C by the hanging-drop vapor-diffusion method in a 1∶1 (v/v) ratio of protein and reservoir, as follows. (1) HA70 was crystallized with a reservoir solution composed of 0.1 M sodium acetate (pH 4.4) and 1.5 M ammonium chloride. Carbohydrate complexes were obtained when HA70 crystals were soaked in the mother liquor supplemented with 100 mM α2,3-SiaLac, α2,6-SiaLac, or Neu5Ac at 18°C overnight. (2) The HA17–HA33 complex was crystallized using a reservoir of 0.1 M MES (pH 6.2), 0.1 M MgCl_2_, and 5% (w/v) PEG [poly(ethylene glycol)] 8K. Micro-seeding was used to improve crystal quality. Carbohydrate complexes were obtained when crystals of the HA17–HA33 complex were soaked with 100 mM Gal, Lac, or LacNAc at 18°C overnight. (3) The HA70^D3^–HA17 complex was crystallized using 0.1 M sodium acetate (pH 4.8), 12% (w/v) PEG MME 2K, and 0.1 M CsCl. (4) The HA70^D3^–HA17–HA33 complex was crystallized using 0.1 M Tris (pH 8.2), 0.1 M NaCl, and 6% (w/v) PEG 20K.

### Diffraction data collection

The crystals of HA70 and its carbohydrate complexes were cryoprotected in their original mother liquor supplemented with 20% (v/v) ethylene glycerol and flash-frozen in liquid nitrogen. Crystals for all the other samples were cryoprotected in 22% (v/v) glycerol with their mother liquors and flash-frozen in liquid nitrogen. X-ray diffraction data were collected at the Stanford Synchrotron Radiation Lightsource (SSRL) or Advanced Photon Source (APS). The data were processed with HKL2000 [57] or iMOSFLM [58]. Data collection statistics are summarized in Tables S2 and S4 in Text S1.

### Structure determination

The structure of HA70 of BoNT/A was determined by molecular replacement software Phaser [59] using the HA70 of BoNT/C (PDB code 2ZS6) [15] as the search model. The D3 domain of HA70 of BoNT/A, together with HA17 of BoNT/D (PDB code 2E4M) [14] and HA33 of BoNT/A (PDB code 1YBI) [17], were used as the search models to solve the structure of the HA70^D3^–HA17–HA33 complex by Phaser. The structures of the HA17–HA33 and the HA70^D3^–HA17 complexes were determined by Phaser using partial structures of the HA70^D3^–HA17–HA33 complex as search models.

The manual model building and refinements were performed in COOT [60] and PHENIX [61] in an iterative manner. The carbohydrates were modeled into the corresponding structure during the refinement based on the Fo-Fc electron density maps. The refinement progress was monitored with the free R value using a 5% randomly selected test set [62]. The structures were validated through the MolProbity web server [63] and showed excellent stereochemistry. Structural refinement statistics are listed in Tables S2 and S4 in Text S1. The coordinate and diffraction data for all the structures reported here will be deposited in the Protein Data Bank. The conformational change of HA33 was measured by DynDom [64]. All structure figures were prepared with PyMol (http://www.pymol.org).

### Isothermal titration calorimetry (ITC)

The calorimetry titration experiments were performed at 23°C on an ITC200 calorimeter from Microcal/GE Life Sciences (Northampton, MA). The HA samples were used as the titrand in the cell and the carbohydrates were used as titrants in the syringe. To control for heat of dilution effects, protein samples were dialyzed extensively against the titration buffer (50 mM Tris, pH 7.6, and 100 mM NaCl) prior to each titration. Carbohydrates and nLoop peptide were dissolved in the same buffer. The pH of the acidic Neu5Ac solution was carefully adjusted to pH 7.6. The following concentrations were used for pair-wise titrations: HA33 (200 µM) vs. carbohydrates (Gal, Lac, LacNAc, IPTG, or α2,6-SiaLac) (50 mM); HA70^D3^ (200 µM) vs. α2,3-, or α2,6-SiaLac (40 mM); HA70^D3^ (160 µM) vs. Neu5Ac (80 mM); and HA70 (30 µM) vs. nLoop (400 µM). The data were analyzed using the Origin software package provided by the ITC manufacturer. The thermodynamic values reported are the average of three independent experiments (Table S3 in Text S1).

### Proteolysis with trypsin or pepsin

The recombinant HA70–HA17–HA33 complex, HA70, the HA17–HA33 complex, and the M-PTC were subjected to limited proteolysis with trypsin and pepsin overnight at room temperature. The trypsin digestions were performed at two different pHs in buffers containing 50 mM sodium phosphate (pH 6.0 or 7.5) and 300 mM NaCl, or in the Krebs-Ringer's solution (119 mM NaCl, 2.5 mM KCl, 1.0 mM NaH_2_PO_4_, 2.5 mM CaCl_2_, 1.3 mM MgCl_2_, 20 mM Hepes, and 11 mM D-glucose). The trypsin∶sample ratios (w/w) were 1∶10 (pH 6.0) or 1∶20 (pH 7.5). The digestions were stopped by adding 1 mM PMSF and boiling the samples in reducing SDS-loading buffer for 10 minutes. The pepsin digestions were performed at a 1∶100 ratio (w/w) of pepsin∶sample in a buffer containing 50 mM citrate acid (pH 2.6, an optimal pH for the pepsin reaction) and 300 mM NaCl. Pepsin digestions were terminated by addition of a 1 M Tris-HCl (pH 8.0) stock solution to give a final concentration of 200 mM and samples were then boiled in the reducing SDS-loading buffer. All samples were subjected to SDS-PAGE.

### Transwell assay

Cell culture: Caco-2 cells were obtained from the German Cancer Research Center (Heidelberg, Germany). Cells were cultured in Dulbecco modified Eagle medium (DMEM, Gibco® | Life Technologies, Darmstadt) supplemented with 10% fetal bovine serum, 100 U of penicillin per ml, and 100 mg of streptomycin per ml for up to six months. The cells were subcultured twice a week and seeded on BD Falcon Cell Culture Inserts (#353494, growth area 0.9 cm^2^, pore size 0.4 µm) at a density of approximately 10^5^ cells cm^−2^ for flux studies and determination of transepithelial electrical resistance (TER).

Measurement of TER: All TER experiments were conducted in 0.5 ml and 1.5 ml of Iscoves Modified Dulbeccos Medium without phenol red (IMDM, Gibco® | Life Technologies, Darmstadt) in the apical and basolateral reservoir, respectively. TER was determined with an epithelial volt-ohm meter (World Precision Instruments, Berlin, Germany) equipped with an Endohm 12 chamber for filter inserts. Filters with cell monolayers were used at day 11 after seeding which is seven days of post confluency. Only filters with an initial resistance of ≥300 Ω cm^−2^ were used. For analysis of independent experiments subsequent results were expressed as percentages of the corresponding resistance of each data set determined immediately after administration of samples. Values are expressed as means of ≥3 independent experiments with duplicate samples ± standard deviations.

Carbohydrate inhibition assays: Lac, Gal, IPTG, Neu5Ac, α2,6- and α2,3-SiaLac were dissolved in IMDM, sterile filtered and stored at −20°C. Neu5Ac stock solution was adjusted to pH 7.4. The wild type HA complex (HA wt), fluorescence-labeled HA complex (HA*), or the HA^70-TPRA^ complex were pre-incubated with the corresponding carbohydrate over night at 4°C in IMDM and diluted to the final concentration with IMDM prior to administration. The TER upon administration of each carbohydrate in the highest concentrations used was checked in the absence of HA and was virtually identical to that of the control without sugars.

Transport measurement: For paracellular transport studies, filters were incubated in IMDM added to the apical (0.5 ml) and basolateral (1.5 ml) reservoir. As marker substance Alexa Fluor® 488 labeled HA* or HA^33-DAFA^* was administered to the apical or basolateral reservoirs at final concentrations of 58 nM and 17 nM, respectively. After 24 hour of incubation, 200 µl of samples were taken from the apical and the basolateral reservoir. The marker substance was measured in a BioTek Synergy 4 fluorescence spectrophotometer at 495 nm excitation and 519 nm emission wavelengths.

### Mouse protection assay

The mouse protection assay was performed following a previously described protocol [4]. Briefly, random sets of 10–20 female Swiss Webster mice (20–23 g) were used per dose. Mice were treated by oral gavage with 100 µl containing 1.9 µg of L-PTC/A (Metabiologics) in phosphate–gelatin buffer (10 mM phosphate buffer, pH 6.2, and 2% gelatin), with or without the indicated concentrations of IPTG, Neu5Ac, or Gal. Mice were also administered 100 µl of 500 mM IPTG by gavage 1 hour prior or after treatment with 100 µl containing 1.9 µg of L-PTC/A in phosphate gelatin buffer. The acidic Neu5Ac was adjusted to pH 6.2 for administration. Mice were monitored for botulism symptoms for up to 14 days post-intoxication. Median survival and *p*-values were determined with the GraphPad Prism 5 program (San Diego, CA).

### Accession numbers

Atomic coordinates and structure factors for HA70, HA70^D3^–HA17–HA33, HA70^D3^–HA17, HA17–HA33, HA17–HA33–Lac, HA17–HA33–Gal, HA17–HA33–LacNAc, HA70–α2,3-SiaLac, and HA70–α2,6-SiaLac have been deposited with the Protein Data Bank under accession codes 4LO4, 4LO7, 4LO8, 4LO0, 4LO2, 4LO1, 4LO3, 4LO5, 4LO6, respectively. EM 3D reconstructions for the L-PTC and the HA complex have been deposited with the Electron Microscopy Data Bank (EMDB) under accession codes EMD-2417 and EMD-2416, respectively.

## Supporting Information

Text S1 **File contains Figures S1–S16 and Tables S1–S4, with legends.**
(PDF)
