# Supplementary material for: Structure of a Bimodular Botulinum Neurotoxin Complex Provides Insights into Its Oral Toxicity
Source: PLoS Pathog. 2013 Oct 10;9(10):e1003690. doi: 10.1371/journal.ppat.1003690 (PMC3795040; doi:10.1371/journal.ppat.1003690)
Supplement: Text S1 — File contains Figures S1–S16 and Tables S1–S4, with legends. (PDF) [file ppat.1003690.s001.pdf]

Text S1: Supporting Information

Structure of a bimodular botulinum neurotoxin complex provides insights into its oral toxicity

Kwangkook Lee[1,7], Shenyan Gu[1,7], Lei Jin[2], Thi Tuc Nghi Le[3], Luisa W. Cheng[4], Jasmin Strotmeier[3], Anna Magdalena Kruel[3], Guorui Yao[1], Kay Perry[5], Andreas Rummel[3,*], Rongsheng Jin[1,6,*]

[1]Department of Physiology and Biophysics, University of California, Irvine, CA 92697, USA.

[2]Infectious and Inflammatory Disease Center, Sanford-Burnham Medical Research Institute, 10901 North Torrey Pines Road, La Jolla, CA 92037, USA.

[3]Institut für Toxikologie, Medizinische Hochschule Hannover, Carl-Neuberg-Str. 1, 30625 Hannover, Germany.

[4]Foodborne Contaminants Research Unit, Western Regional Research Center, U.S. Department of Agriculture, Agricultural Research Service, 800 Buchanan Street, Albany, CA 94710, USA.

[5]NE-CAT and Department of Chemistry and Chemical Biology, Cornell University, Building 436E, Argonne National Laboratory, 9700 S. Cass Avenue, Argonne, IL 60439, USA.

[6]Neuroscience, Aging and Stem Cell Center, Sanford-Burnham Medical Research Institute, 10901 North Torrey Pines Road, La Jolla, CA 92037, USA

[7]These authors contributed equally to this work.

[*]Correspondence should be addressed to R.J. (r.jin@uci.edu) or A.R. (rummel.andreas@mh-hannover.de)

This file includes

Figures S1–S16

Tables S1–S4

Supporting References

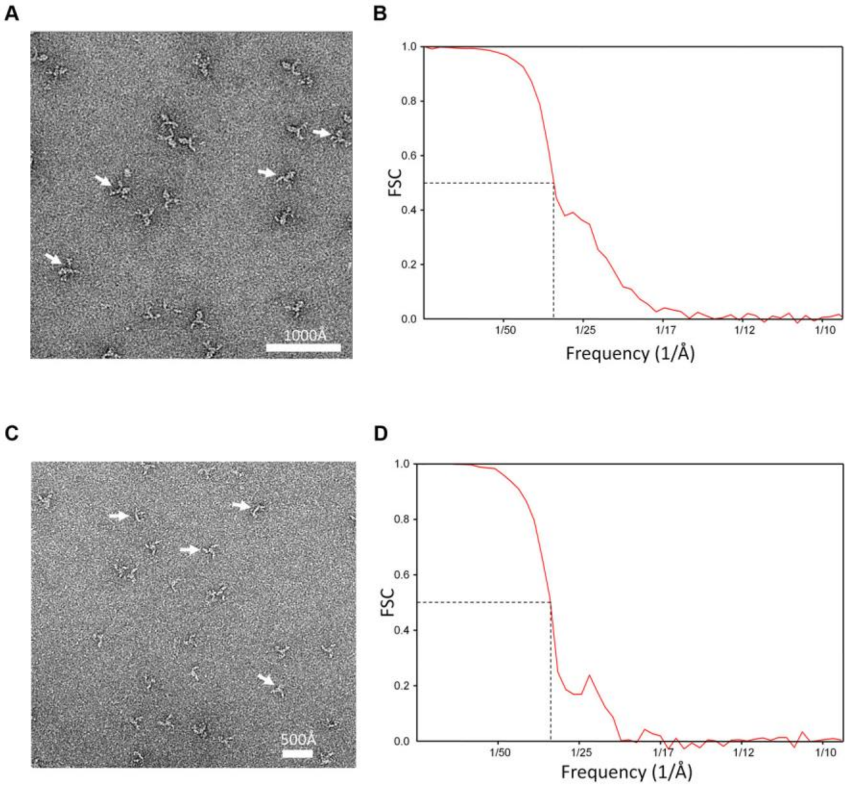

Figure S1. Electron microscopy of L-PTC/A and the recombinant HA complex. (A) A representative negative-staining raw image for L-PTC/A. Arrows indicate particles of four different orientations. Scale bar = 1,000 Å. (B) Fourier shell correlation (FSC) curve as a function of resolution for L-PTC/A 3D reconstruction. Reported resolution at ~30.8 Å is based on a cutoff value of FSC = 0.5 (dashed horizontal/vertical lines). (C) A representative negative-staining raw image for the HA complex. Arrows indicate particles of four different orientations. Scale bar = 500 Å. (D) FSC curve as a function of resolution for the HA complex 3D reconstruction, which indicates a resolution at ~30.6 Å.

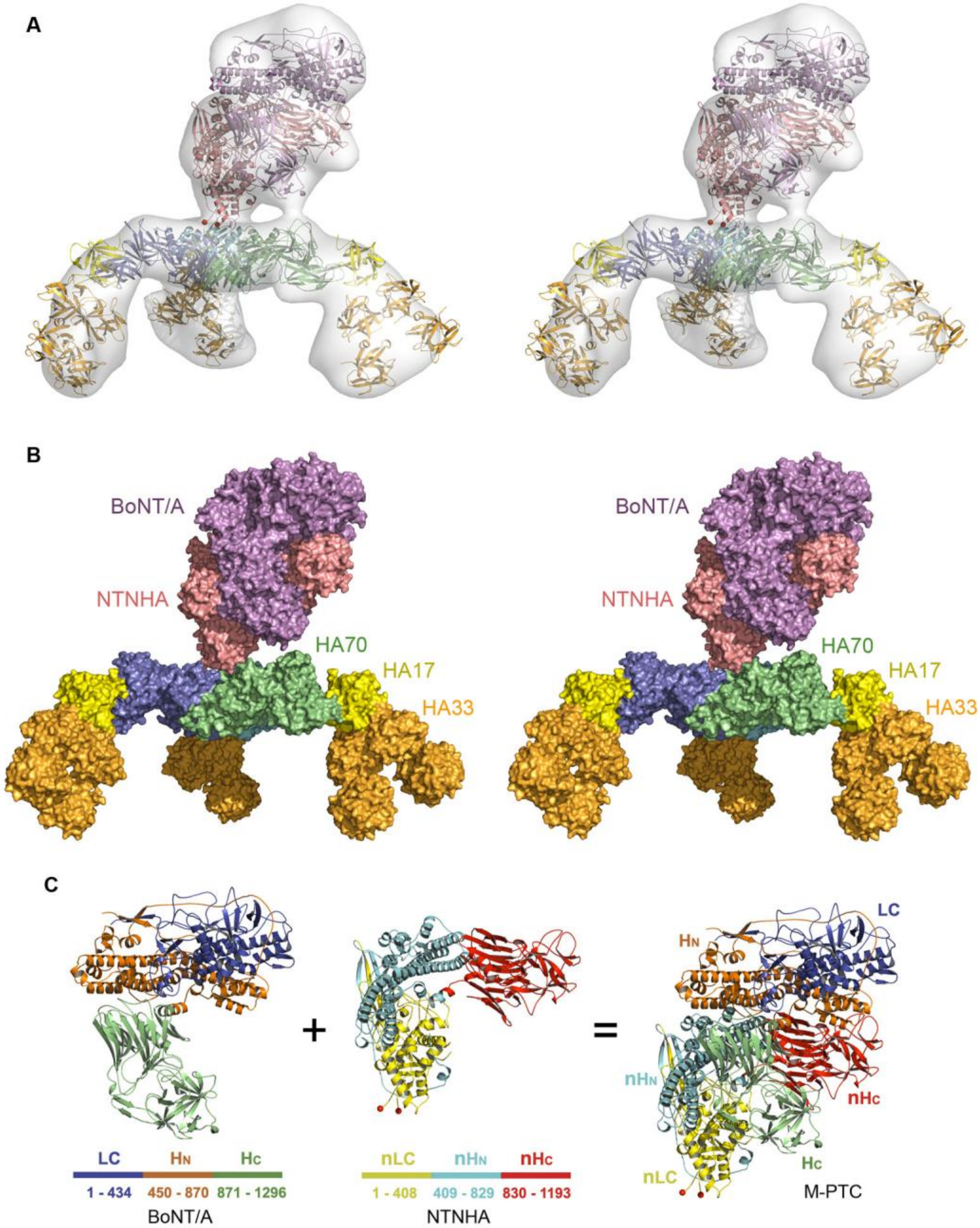

Figure S2. Structure of L-PTC/A. L-PTC/A is orientated and colored in the same manner as for Fig. 1. (A) A stereo view of the 3D-EM reconstruction of L-PTC/A. (B) A stereo view of L-PTC/A in surface representation. (C) The domain organization of the M-PTC, which is in the same orientation as that in the context of L-PTC/A shown in (A) and (B) [1].

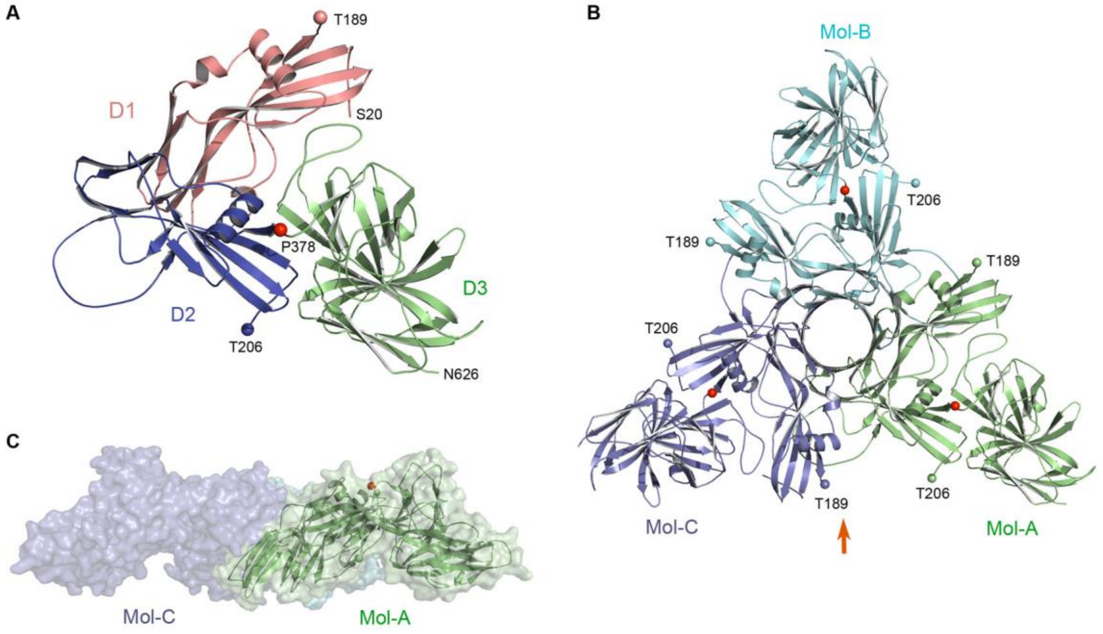

Figure S3. Structure of HA70 at 2.9 Å resolution. (A) HA70 is composed of three domains: D1 (residues M1–T189, salmon), D2 (T206–D377, blue), and D3 (P378–N626, green). The linker between D1 and D2 (T190–S205) and the N-terminal 19 residues are not visible in the crystal structure. (B) HA70 forms a homo-trimer, mediated by D1 and D2. The red arrow indicates the viewing direction of (C). (C) A side view of the HA70 trimer in surface representation, with Mol-A of HA70 shown as ribbons.

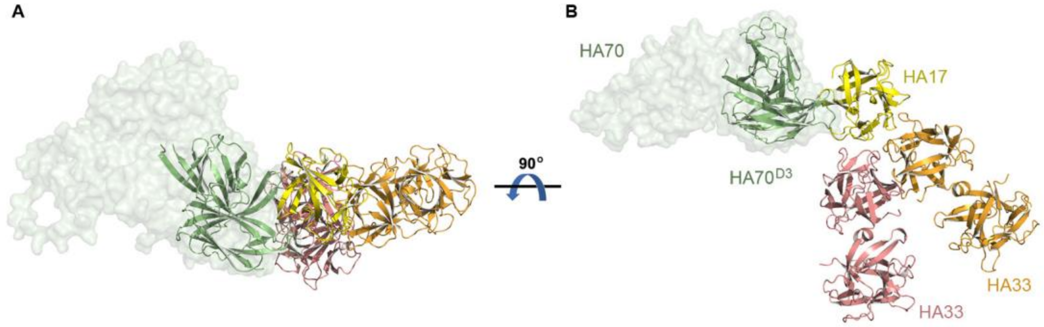

Figure S4. Structure of the mini-HA complex (HA70^{D3}–HA17–HA33) at 3.7 Å resolution. (A, B) The mini-HA complex is composed of one HA70^{D3} (P378–N626, green), one HA17 (yellow), and two HA33s (orange or salmon). The structure of HA70^{D3} superimposes well with the full-length HA70, which is shown as a transparent surface representation. The two views differ by a 90° rotation, as indicated. The orientations in (A) and (B) are similar to those shown in Fig. S3B and S3C, respectively.

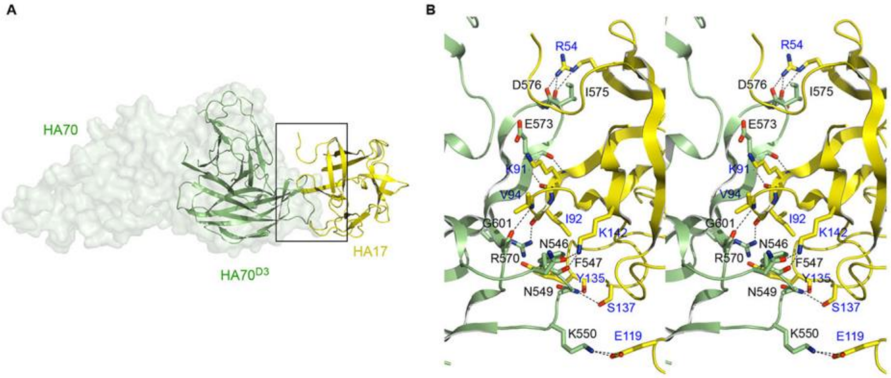

Figure S5. Structure of the HA70^{D3}–HA17 complex at 2.4 Å resolution. (A) The structure of HA70^{D3} (green ribbon) superimposes well with D3 of the full-length HA70 (green surface). Open-book view of the interface highlighted in the box is shown in (B). (B) A stereo view of the interface between HA70^{D3} and HA17. Key interacting residues are shown as sticks, while the residues in HA70^{D3} and HA17 are labeled in black and blue, respectively. Hydrogen bonds and salt bridges are indicated by black dashed lines.

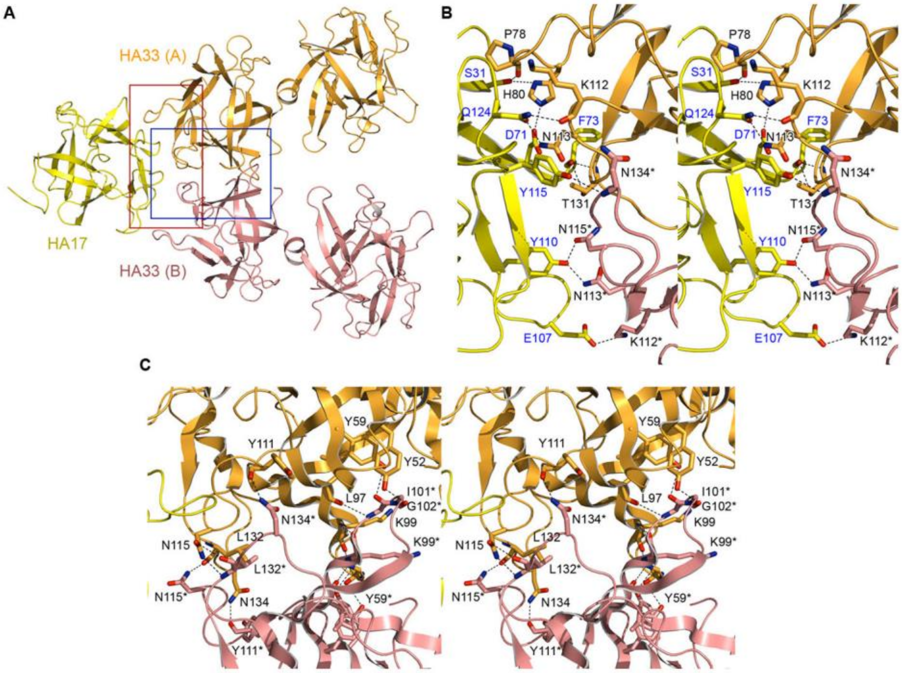

Figure S6. Structure of the HA17–HA33 complex at 2.1 Å resolution. (A) HA17 (yellow) simultaneously binds to two HA33s (Mol-A in orange and Mol-B in salmon). Open-book views of the interfaces highlighted in red and blue boxes are shown in (B) and (C), respectively. Key interacting residues are shown as sticks. The residues in HA17, HA33-Mol-A, and HA33-Mol-B are labeled as blue, black, and black with an asterisk, respectively. Hydrogen bonds and salt bridges are indicated by black dashed lines. (B) A stereo view of the interface between HA17 and HA33. (C) A stereo view of the interface between HA33 Mol-A and Mol-B. Due to the 2-fold symmetry of the two HA 33 molecules, intra-HA33 interactions are symmetric, except for Asn115.

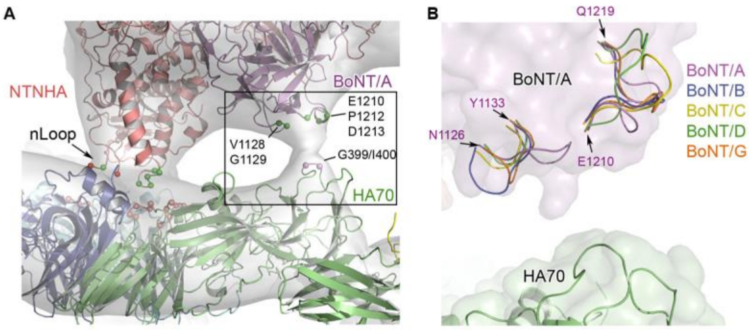

Figure S7. A close-up view of the interface between the M-PTC and the HA complex. (A) The L-PTC is oriented similarly to that shown in Fig. 1A. The Cα atoms of the potential interacting residues are shown as spheres. The box highlights the interface formed between BoNT/A-H_C and HA70. (B) The structures of the H_C of BoNT/B, C, D, and G are superimposed with BoNT/A in the M-PTC [2-5]. BoNT/E and BoNT/F are not included in the analysis because they are encoded in gene clusters that lack HA genes. For clarity, only two loop regions are shown: BoNT/A (N1126–Y1133 and E1210–Q1219), BoNT/B (K1113–V1118 and P1197–N1206), BoNT/C (N1127–Q1130 and E1205–I1214), BoNT/D (E1114–V1117 and S1195–G1203), and BoNT/G (F1121–M1126 and P1205–D1214). The loop boundaries of BoNT/A are labeled. BoNT/A and HA70 in the L-PTC are shown as transparent surface representations.

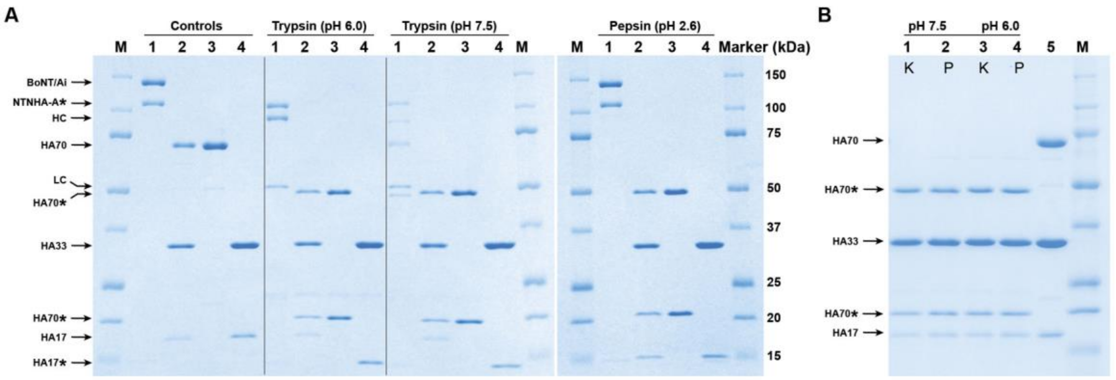

Figure S8. The HAs are stable at acidic pH and are resistant to digestive proteases. (A) The recombinant M-PTC (lane 1), the HA complex (HA70–HA17–HA33, lane 2), HA70 (lane 3), and the HA17–HA33 complex (lane 4) were treated with trypsin at pH 6.0 or pH 7.5, or with pepsin at pH 2.6 (optimal pH for pepsin cleavage). (B) The recombinant HA complex was treated with trypsin at pH 6.0 or pH 7.5 using a phosphate buffer (P) or the physiological Krebs-Ringer's solution (K). Lane 5 is the control sample without trypsin. No difference was observed between the two buffers. The HA70 is nicked into two peptide chains of ~22 kDa and ~48 kDa (HA70*), which is reminiscent of the post-translational nicking of HA70 that occurs physiologically [6]. HA17 could be nicked into a ~15 kDa peptide (HA17*) [7].

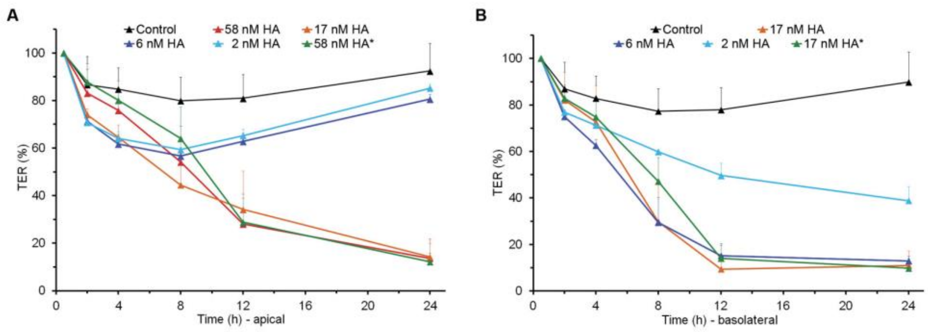

Figure S9. The fully assembled HA complex markedly reduced the TER of Caco-2 cell monolayers. Caco-2 cells were grown on transwell filter membranes into confluent polarized monolayers. TER was measured when various concentrations of the HA complex was applied to the apical (A) or basolateral (B) chambers. The HA complex containing Alexa-488 labeled HA70 (HA*) has the same potency as the unlabeled HA complex (HA). Values are means ± SD (n = 4–12).

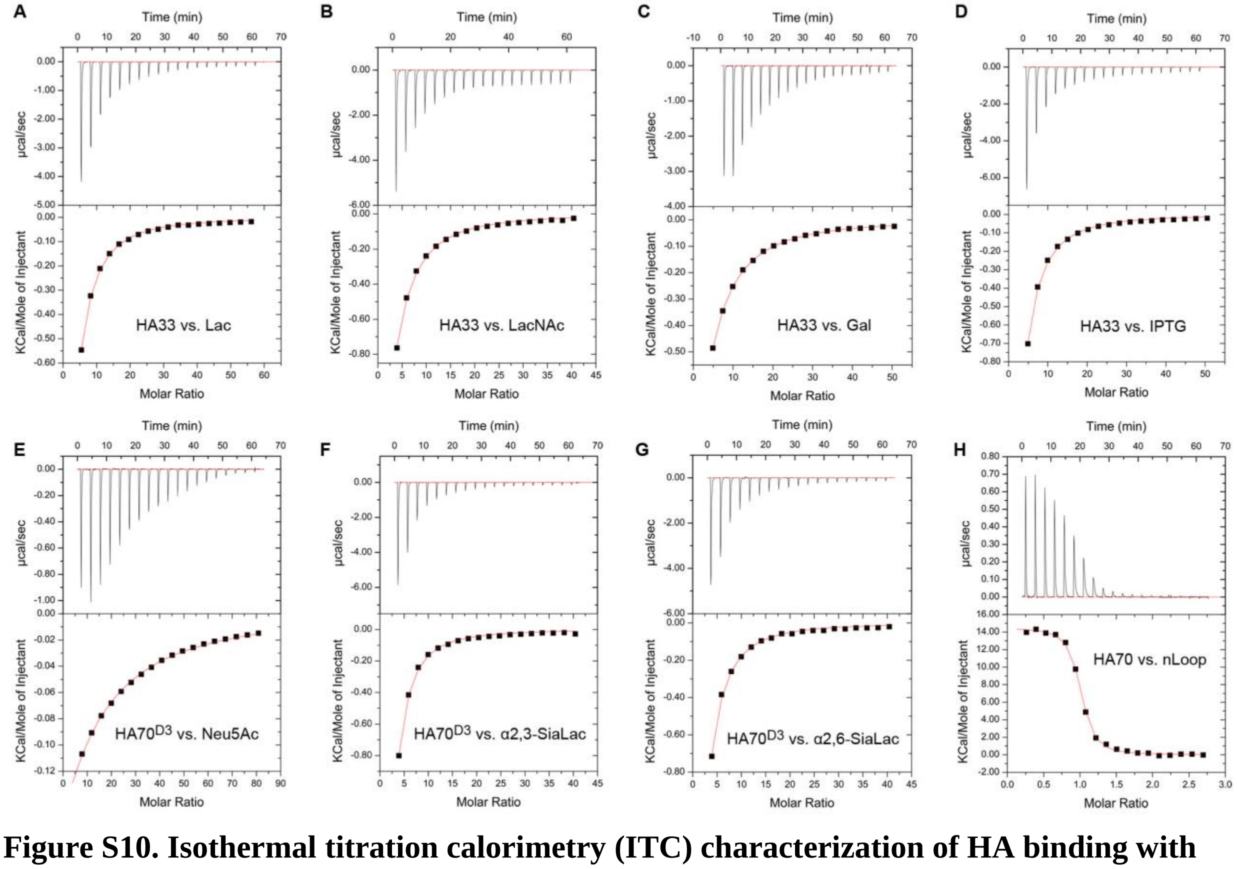

Figure S10. Isothermal titration calorimetry (ITC) characterization of HA binding with carbohydrate receptors or NTNHA-A. The representative ITC data are shown for (A) HA33 vs. Lac. (B) HA33 vs. LacNAc. (C) HA33 vs. Gal. (D) HA33 vs. IPTG. (E) HA70^{D3} vs. Neu5Ac. (F) HA70^{D3} vs. α2,3-SiaLac. (G) HA70^{D3} vs. α2,6-SiaLac. (H) HA70 vs. nLoop (G116–A148) of NTNHA-A.

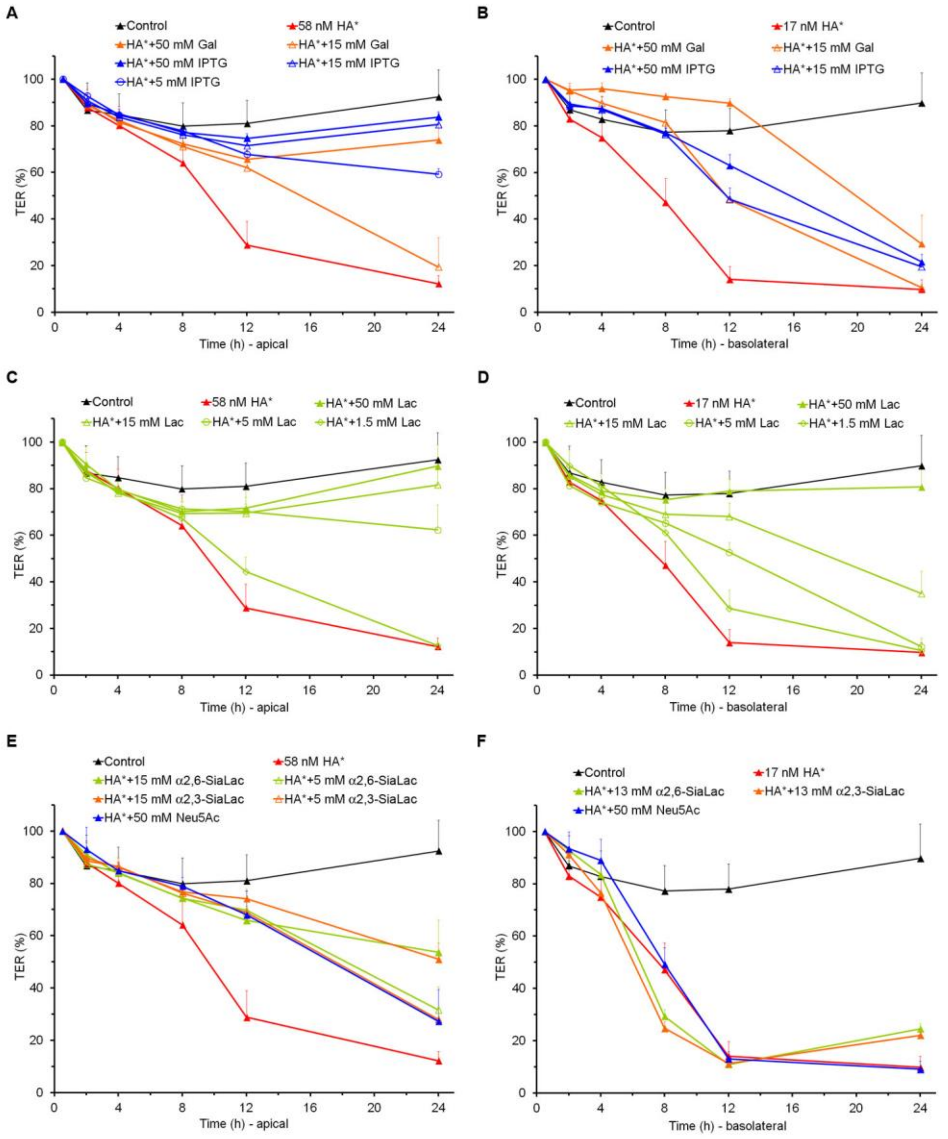

Figure S11. Carbohydrate receptor mimics inhibited the reduction of TER induced by the HA complex. Caco-2 cells were grown on transwell filter membranes into confluent polarized monolayers. (A, B) TER was measured when fluorescence-labeled HA complex (HA*) pre-incubated with Gal or IPTG was applied to the apical (A; 58 nM) or basolateral (B; 17 nM) chambers. (C, D) TER was measured when HA* pre-incubated with Lac was applied to the apical (C; 58 nM) or basolateral (D; 17 nM) chambers. (E, F) TER was measured when HA* pre-incubated with α2,3-SiaLac, α2,6-SiaLac, or Neu5Ac was applied to the apical (E; 58 nM) or basolateral (F; 17 nM) chambers. Values are means ± SD (*n* = 4–12).

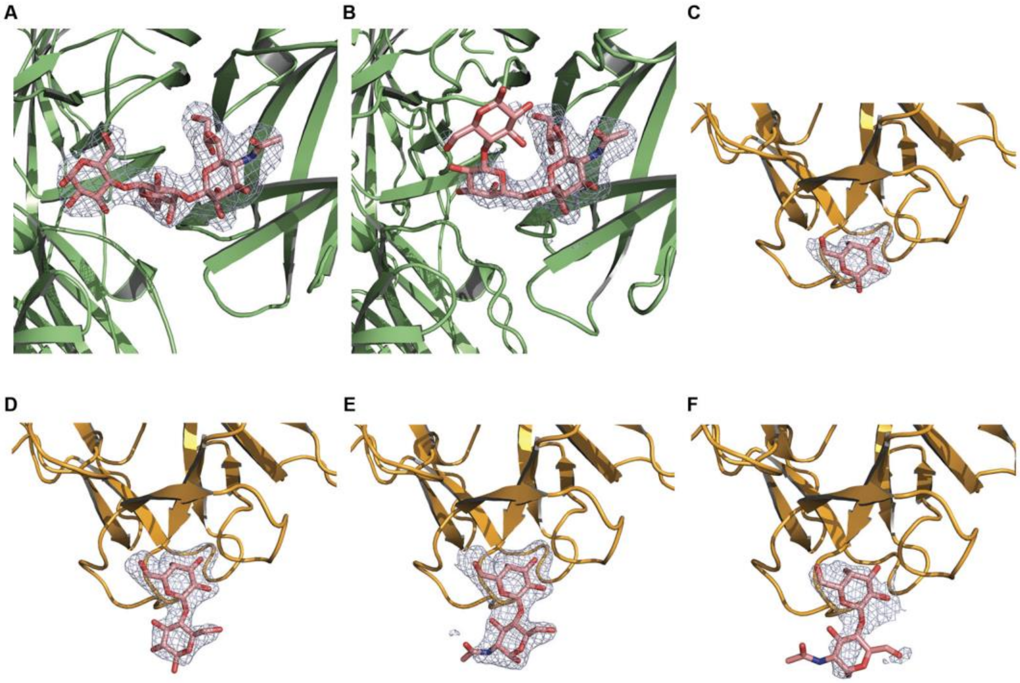

Figure S12. F_O-F_C omit electron density maps around the bound carbohydrates, overlaid with the final model. HA70 and HA33 are in green and orange, respectively. (A) HA70–α2,3-SiaLac (contoured at 3.0 σ). (B) HA70–α2,6-SiaLac (contoured at 3.0 σ). There are two HA33 molecules in one symmetry unit (chain A and B). For Lac and LacNAc that bind to chain A, Glc-O5 and GlcNAc-O5 forms a hydrogen bond with Ser80-OG of HA17 in the neighboring AU. There is no crystal contact involving both molecules of Gal in the AU or the Lac and LacNAc molecule that binds to HA33-chain B. (C) HA33(chain A)–Gal (contoured at 3.5 σ). (D) HA33 (chain A)–Lac (contoured at 2.5 σ). (E) HA33(chain A)–LacNAc (contoured at 2.5 σ). (F) HA33(chain B)–LacNAc (contoured at 2.5 σ).

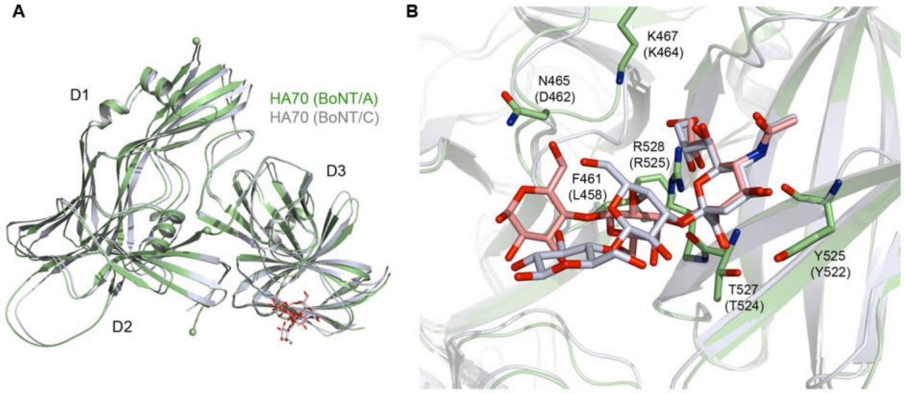

Figure S13. HA70-A (green) and HA70-C (gray) adopt a similar structure. (A) HA70-A and HA70-C [8,9] were superimposed based on 582 Cα atoms yielding an rmsd of 1.43 Å. (B) A close-up view of the receptor-binding pocket in HA70. α2,3-SiaLac is shown in pink or gray when bound to HA70-A or HA70-C, respectively. The binding mode for the terminal Neu5Ac is almost identical in the two structures. The Neu5Ac-binding residues in HA70-A are shown as sticks, with the equivalent residues in HA70-C in parentheses. The key Neu5Ac-binding residues in HA70 (e.g. Tyr525, Thr527, Arg528, and Lys467) are highly conserved among BoNT/A, B, C, and D (HA70-G was not available for analysis, and BoNT/E and BoNT/F gene clusters do not encode HAs), suggesting the binding mode is conserved.

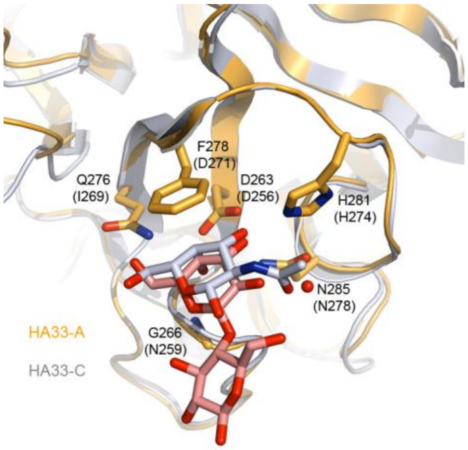

Figure S14. HA33-A and HA33-C show similar modes of binding to galactose. The structures of the HA33-A–Lac complex (orange/pink) and the HA33-C–Gal complex (gray/gray) [10] were superimposed based on Cα atoms in the C-terminal domain of HA33 (rmsd of ~1.19 Å over 134 Cα atoms). Key Gal-binding residues in HA33-A are shown as sticks, with the equivalent residues in HA33-C in parentheses. The F278 residue in HA33-A that provides the crucial stacking interaction with Gal is replaced with D271 in HA33-C.

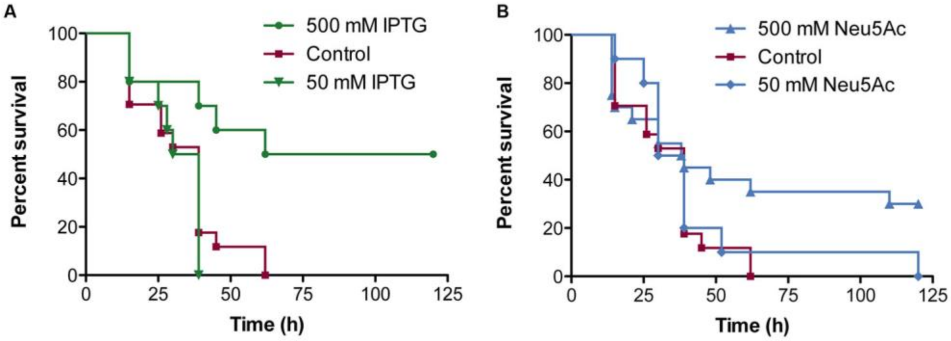

Figure S15. Survival time comparisons of mice. Mice were treated orally with L-PTC/A in the presence of IPTG (A) and Neu5Ac (B).

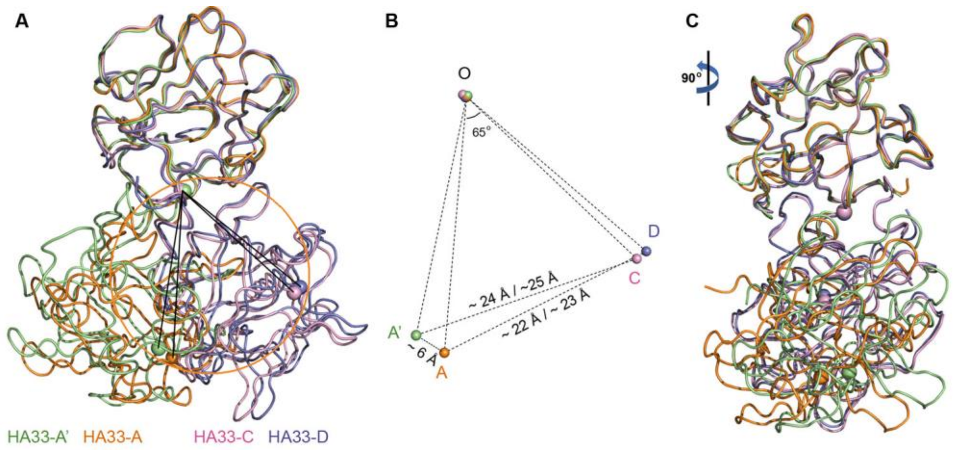

Figure S16. HA33 adopts a flexible conformation. (A) The structures of HA33 of BoNT/A in the current work (HA33-A, orange) and that from an earlier publication (HA33-A', green) [11], HA33 of BoNT/C (HA33-C, pink) [10], and HA33 of BoNT/D (HA33-D, blue) [12] are superimposed based on Cα atoms in their N-terminal domains. The area highlighted in an orange circle is shown in (B). (B) Residue Asp144 of HA33-A (equivalent to Asn141 in HA33-C/D), which is located in the linker connecting the two HA33 domains, is used as the origin (labeled as O) for analysis. A key Gal-binding residue Asp263 of HA33-A and HA33-A', and its equivalent Asp256 of HA33-C/D (labeled as A, A', C, and D, respectively) were used to quantify the inter-domain twist of HA33. In comparison to HA33-A, HA33-A' twists ~14° and moves ~6 Å; HA33-C twists ~61° and moves ~22 Å; and HA33-D twists ~65° and moves ~23 Å. (C) A view of the superimposed HA33 rotated 90° from that shown in (A).

Table S1. Characterization of the assembly of the HA complex by analytical ultracentrifugation

HA	Composition of the Basic Unit[a]		Oligomerization[a]	K_d (μM)[b]	pH/NaCl (mM)[c]
HA33	HA33	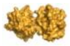	Monomer	N.A.	pH 7.6/100
			Dimer	56.6 ± 15.6[b]	pH 2.3/100
HA70/17	3×HA70 + 3×HA17	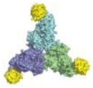	Monomer	N.A.	pH 7.6/100
			N.A.[d]	N.A.[d]	pH 2.3/100[d]
HA17/33	1×HA17 + 2×HA33	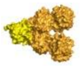	Monomer	N.A.	pH 2.3/100
			Monomer	N.A.	pH 7.6/100
HA70^{D3}/17/33	1×HA70^{D3} + 1×HA17 + 2×HA33	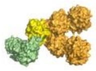	Monomer	N.A.	pH 2.3/100
			Dimer	23.1 ± 2.2[b]	pH 7.6/100
HA70/17/33	3×HA70 + 3×HA17 + 6×HA33	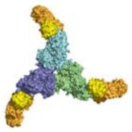	Monomer	N.A.	pH 2.3/100
			Dimer	10.9 ± 3.4[b]	pH 7.6/100
			Monomer	N.A.	pH 7.6/500

[a] The basic unit is the predominant form observed in solution and is defined as the "monomer". "Dimer" represents dimerization of the basic unit.

[b] The weak dissociation constant (K_d) suggests that further dimerization of the basic unit is unlikely to occur under physiological conditions.

[c] pH 2.3 buffer: 50 mM citric acid; pH 7.6 buffer: 50 mM Tris-HCl.

[d] The HA70–HA17 complex precipitated at pH 2.3.

Table S2. Data collection and refinement statistics for the HA complexes

	HA70	HA70^{D3}–HA17–HA33	HA70^{D3}–HA17	HA17–HA33
**Data collection**				
Space group	$I4_132$	$P2_12_12$	$P2_1$	$C222_1$
Cell dimensions				
a, *b*, *c* (Å)	261.7, 261.7, 261.7	159.6, 187.5, 120.4	45.8, 228.9, 78.7	106.6, 118.7, 162.5
α, β, γ (º)	90, 90, 90	90, 90, 90	90, 92.1, 90	90, 90, 90
Resolution (Å)	50–2.9 (3.0–2.9)	50–3.7 (3.9–3.7)	50–2.4 (2.5–2.4)	47.9–2.1 (2.2–2.1)
R_{merge}	8.3 (75.7)	11.9 (65.6)	12.5 (68.9)	4.9 (59.1)
$I/\sigma I$	14.8 (2.3)	7.4 (2.1)	8.4 (1.7)	13.7 (2.0)
Completeness (%)	99.9 (100)	99.7 (100)	99.5 (99.0)	98.1 (97.5)
Redundancy	7.0 (6.6)	3.6 (3.7)	4.7 (4.5)	3.7 (3.6)
**Refinement**				
Resolution (Å)	44.9–2.9	49.2–3.7	45.8–2.4	35.6–2.1
No. reflections	34,988	38,136	62,548	62,896
$R_{work/}$ R_{free}	18.6/20.6	26.9/29.3	19.7/24.9	18.8/21.7
No. atoms				
Protein	4,760	15,330	12,276	5,849
Ligand/ion	1	0	0	0
Water	26	0	413	283
B-factors (Å^2)				
Protein	86.8	165.6	55.9	53.7
Ligand/ion	69.6			
Water	60.3		40.4	46.8
R.m.s. deviations				
Bond lengths (Å)	0.005	0.009	0.003	0.010
Bond angles (º)	0.913	1.127	0.760	1.214

* One crystal was used for each structure.
* Values in parenthesis are for highest-resolution shell.

Table S3. ITC characterization of HA binding with the carbohydrate receptors and NTNHA-A

Interactions between HAs and carbohydrates

HA	Carbohydrate	K_d (mM)	ΔH (kcal/mol)	ΔS (cal/mol/K)
HA33	Lac	0.95 ± 0.08	-8.9 ± 0.8	-16.2 ± 2.5
	LacNAc	0.93 ± 0.05	-9.1 ± 0.2	-17.0 ± 0.7
	Gal	1.80 ± 0.30	-8.5 ± 1.7	-16.2 ± 6.3
	IPTG	0.80 ± 0.04	-9.2 ± 1.0	-16.9 ± 3.4
	α2,6-SiaLac	No binding		
HA33-D263A	Lac	No binding		
HA33-F278A	Lac	No binding		
$HA70^{D3}$	Neu5Ac	7.77 ± 1.54	-6.0 ± 1.0	-10.7 ± 3.8
	α2,3-SiaLac	0.54 ± 0.23	-8.3 ± 0.1	-13.0 ± 0.5
	α2,6-SiaLac	0.51 ± 0.10	-7.9 ± 0.7	-11.6 ± 2.3
$HA70^{D3}$-T527P	α2,6-SiaLac	No binding		
$HA70^{D3}$-R528A	α2,6-SiaLac	No binding		
HA70-T527P/R528A	α2,6-SiaLac	No binding		

Interactions between HA70 and NTNHA-A

	NTNHA-A	K_d (μM)	ΔH (kcal/mol)	ΔS (cal/mol/K)
HA70	nLoop (G116–A148)	0.34 ± 0.09	11.4 ± 2.7	68.0 ± 9.6

TableS4. Data collection and refinement statistics for the HA–glycan complexes

	HA17–HA33–Lac	HA17–HA33–Gal	HA17–HA33–LacNAc	HA70–α2,3-SiaLac	HA70–α2,6-SiaLac
**Data collection**					
Space group	$C222_1$	$C222_1$	$C222_1$	$I4_132$	$I4_132$
Cell dimensions					
a, *b*, *c* (Å)	106.5, 119.2, 163.1	107.1, 118.7, 162.5	107.2, 118.8, 162.5	260.7, 260.7, 260.7	261.5, 261.5, 261.5
α, β, γ (º)	90, 90, 90	90, 90, 90	90, 90, 90	90, 90, 90	90, 90, 90
Resolution (Å)	50–2.3 (2.4–2.3)	47.9–2.3 (2.4-2.3)	50–2.3 (2.4–2.3)	47.6–2.7 (2.9–2.7)	47.7–2.3 (2.4–2.3)
R_{merge}	5.7 (38.4)	5.4 (60.3)	5.5 (45.5)	7.5 (63.6)	11.0 (57.5)
$I/\sigma I$	14.4 (2.0)	14.5 (1.9)	12.5 (2.2)	12.0 (2.2)	7.8 (1.9)
Completeness (%)	97.4 (98.9)	99.8 (99.9)	99.7 (99.7)	99.9 (100)	99.6 (100)
Redundancy	4.6 (4.3)	3.6 (3.7)	6.4 (6.4)	5.1 (5.3)	7.0 (6.4)
**Refinement**					
Resolution (Å)	32.1–2.3	36.2–2.3	47.9–2.3	44.7–2.7	44.8–2.3
No. reflections	47,909	48,977	49,357	41,439	66,713
R_{work}/R_{free}	16.7/21.1	19.9/22.7	17.9/21.3	19.0/20.8	18.6/21.0
No. atoms					
Protein	5,857	5,849	5,849	4,760	4,760
Ligand/ion	46	24	52	44	44
Water	225	236	217	112	284
B-factors (Å^2)					
Protein	53.3	59.8	54.8	70.8	62.7
Ligand/ion	96.4	83.3	102.7	70.7	90.2
Water	49.8	49.0	46.2	56.9	60.4
R.m.s. deviations					
Bond lengths (Å)	0.008	0.003	0.009	0.003	0.007
Bond angles (º)	1.049	0.768	1.088	0.797	1.063

* One crystal was used for each structure.
* Values in parenthesis are for highest-resolution shell.

Supporting References:

1. Gu S, Rumpel S, Zhou J, Strotmeier J, Bigalke H, et al. (2012) Botulinum neurotoxin is shielded by NTNHA in an interlocked complex. Science 335: 977-981.
2. Jin R, Rummel A, Binz T, Brunger AT (2006) Botulinum neurotoxin B recognizes its protein receptor with high affinity and specificity. Nature 444: 1092-1095.
3. Strotmeier J, Gu S, Jutzi S, Mahrhold S, Zhou J, et al. (2011) The biological activity of botulinum neurotoxin type C is dependent upon novel types of ganglioside binding sites. Mol Microbiol 81: 143-156.
4. Strotmeier J, Lee K, Volker AK, Mahrhold S, Zong Y, et al. (2010) Botulinum neurotoxin serotype D attacks neurons via two carbohydrate-binding sites in a ganglioside-dependent manner. Biochem J 431: 207-216.
5. Stenmark P, Dong M, Dupuy J, Chapman ER, Stevens RC (2010) Crystal structure of the botulinum neurotoxin type G binding domain: insight into cell surface binding. J Mol Biol 397: 1287-1297.
6. Inoue K, Fujinaga Y, Watanabe T, Ohyama T, Takeshi K, et al. (1996) Molecular composition of Clostridium botulinum type A progenitor toxins. Infect Immun 64: 1589-1594.
7. Cheng LW, Henderson TD, 2nd (2011) Comparison of oral toxicological properties of botulinum neurotoxin serotypes A and B. Toxicon 58: 62-67.
8. Nakamura T, Kotani M, Tonozuka T, Ide A, Oguma K, et al. (2009) Crystal structure of the HA3 subcomponent of Clostridium botulinum type C progenitor toxin. J Mol Biol 385: 1193-1206.
9. Yamashita S, Yoshida H, Uchiyama N, Nakakita Y, Nakakita S, et al. (2012) Carbohydrate recognition mechanism of HA70 from Clostridium botulinum deduced from X-ray structures in complexes with sialylated oligosaccharides. FEBS Lett 586: 2404-2410.
10. Nakamura T, Tonozuka T, Ito S, Takeda Y, Sato R, et al. (2011) Molecular diversity of the two sugar-binding sites of the beta-trefoil lectin HA33/C (HA1) from Clostridium botulinum type C neurotoxin. Arch Biochem Biophys 512: 69-77.
11. Arndt JW, Gu J, Jaroszewski L, Schwarzenbacher R, Hanson MA, et al. (2005) The structure of the neurotoxin-associated protein HA33/A from Clostridium botulinum suggests a reoccurring beta-trefoil fold in the progenitor toxin complex. J Mol Biol 346: 1083-1093.
12. Hasegawa K, Watanabe T, Suzuki T, Yamano A, Oikawa T, et al. (2007) A novel subunit structure of clostridium botulinum serotype D toxin complex with three extended arms. J Biol Chem 282: 24777-24783.
